# Optimization and validation of the DESIGNER preprocessing pipeline for clinical diffusion MRI in white matter aging

**DOI:** 10.1162/imag_a_00125

**Published:** 2024-04-08

**Authors:** Jenny Chen, Benjamin Ades-Aron, Hong-Hsi Lee, Subah Mehrin, Michelle Pang, Dmitry S. Novikov, Jelle Veraart, Els Fieremans

**Affiliations:** Center for Biomedical Imaging (CBI), Center for Advanced Imaging Innovation and Research (CAI^2^R), Department of Radiology, New York University Grossman School of Medicine, New York, NY, United States; Athinoula A. Martinos Center for Biomedical Imaging, Department of Radiology, Massachusetts General Hospital, Charlestown, MA, United States; Harvard Medical School, Boston, MA, United States; John A. Burns School of Medicine, University of Hawai’i at Manoa, Honolulu, HI, USA

**Keywords:** diffusion MRI, DTI, DKI, image artifact, preprocessing

## Abstract

Various diffusion MRI (dMRI) preprocessing pipelines are currently available to yield more accurate diffusion parameters. Here, we evaluated accuracy and robustness of the optimized Diffusion parameter EStImation with Gibbs and NoisE Removal (DESIGNER) pipeline in a large clinical dMRI dataset and using ground-truth phantoms. DESIGNER, a preprocessing pipeline targeting various imaging artifacts in diffusion MRI data, has been modified to improve denoising and target Gibbs ringing for partial Fourier acquisitions. We compared the revised DESIGNER (Dv2) (including denoising, Gibbs removal, correction for motion, echo planar imaging (EPI) distortion, and eddy currents) against the original DESIGNER (Dv1) pipeline, minimal preprocessing (including correction for motion, EPI distortion, and eddy currents only), and no preprocessing on a large clinical dMRI dataset of 524 control subjects with ages between 25 and 75 years old. We evaluated the effect of specific processing steps on age correlations in white matter with diffusion tensor imaging (DTI) and diffusion kurtosis imaging (DKI) metrics. We also evaluated the added effect of minimal Gaussian smoothing to deal with noise and to reduce outliers in parameter maps compared to DESIGNER-v2’s noise removal method. Moreover, Dv2’s updated noise and Gibbs removal methods were assessed using a ground truth dMRI phantom to evaluate accuracy. Results show age correlations of DTI and DKI metrics in white matter were affected by the preprocessing pipeline, causing systematic differences in absolute parameter values and loss or gain of statistical significance. Both in clinical dMRI and ground-truth phantoms, Dv2 pipeline resulted in the smallest number of outlier voxels and improved accuracy in DTI and DKI metrics as noise was reduced and Gibbs removal was improved. Thus, DESIGNER-v2 provides more accurate and robust DTI and DKI parameter maps by targeting common artifacts present in dMRI data acquired in clinical settings, as compared to no preprocessing or minimal preprocessing.

## 1 Introduction

Diffusion MRI (dMRI) has the ability to access in-vivo tissue microstructure (Novikov et al., 2019) noninvasively with sensitivity to healthy development, aging, and disease pathology. Quantitative parameters extracted from dMRI are potential biomarkers for early diagnosis and disease-specific treatment and/or prevention planning (Andica et al., 2020; Billiet et al., 2015; Moura et al., 2019). However, raw dMRI in clinic and clinical research applications are unavoidably contaminated by thermal noise and various image artifacts caused by pulse sequence, gradient hardware, acquisition strategy, and patient movement (Jones & Cercignani, 2010; Le Bihan et al., 2006). All these artifacts propagate into diffusion parameter maps, yielding unreliable results to investigators and clinicians.

Common artifacts present in raw dMRI include distortions caused by eddy currents when applying strong gradients in different directions, motion (Le Bihan et al., 2006), and low signal-to-noise ratio (SNR) due to diffusion weighting and the relatively long echo time (Dietrich et al., 2001; Jones & Cercignani, 2010). Although single-shot echo planar imaging (EPI) is fast and the standard acquisition strategy, it causes EPI distortion resulting from local magnetic field inhomogeneities (Le Bihan et al., 2006). In addition, due to finite sampling in k-space, dMRI suffers from Gibbs ringing in regions with high contrast boundaries (Kellner et al., 2016; Perrone et al., 2015; Veraart, Fieremans, et al., 2016). In particular, clinical dMRI is routinely acquired in partial Fourier acquisition mode, leading to additional ringing after reconstruction via zero filling. All these different sources of artifacts contribute to unphysical dMRI parameter estimates and can appear as unfeasible values (Dubkov & Malakhov, 1976; Henriques, Correia, et al., 2021; Kuder et al., 2012; Olson et al., 2018; Veraart, Fieremans, et al., 2016) in parametric maps.

Various dMRI preprocessing pipelines have been implemented to minimize some or all of these artifacts (Ades-Aron et al., 2018; Cieslak et al., 2021; Cui et al., 2013; Glasser et al., 2013; Irfanoglu et al., 2017; Maximov et al., 2019; Tournier et al., 2019), aiming to improve the accuracy and precision of outcome parameter maps. As numerous artifacts exist, dMRI pre-processing pipelines can range in complexity depending on study protocol and software availability. For example, a minimal preprocessing pipeline from the Human Connectome Project (HCP) (Glasser et al., 2013) corrects for EPI distortion, eddy currents, and motion, while more complex pipelines such as Diffusion parameter EStImation with Gibbs and NoisE Removal (DESIGNER) (Ades-Aron et al., 2018) additionally target removal of common noise and Gibbs ringing artifacts. MRtrix3 (Tournier et al., 2019), TORTOISE (Irfanoglu et al., 2017), and DIPY (Garyfallidis et al., 2014) are among many other software tools that make a range of previously proposed artifact correction methods available to the community and allow users to build a customized pipeline specific to their needs.

It has been observed that the inclusion of these additional preprocessing steps improves the performance of widely used preprocessing steps such as motion and eddy current distortion correction (Cieslak et al., 2024). However, as observed by Maximov et al. (2019), pipeline variations not only affect diffusion metrics, they can possibly result in conflicting findings in the same study. There is a need to evaluate these correction steps to decide which techniques are optimal so we can confidently set up a preprocessing pipeline for future large-scale processing and interpret the study results.

In this study, we revisited the originally proposed DESIGNER (Diffusion parameter EStImation with Gibbs and NoisE Removal) (Ades-Aron et al., 2018) pipeline as a targeted artifact correction pipeline for processing typical dMRI as acquired in a clinical setting. The DESIGNER pipeline is updated here to include improved denoising and Gibbs ringing removal methods suitable for partial Fourier acquisitions. We will call this updated DESIGNER, DESIGNER-v2. To evaluate and demonstrate the impact of varying preprocessing on statistical outcomes and results, we applied DESIGNER-v2, minimal preprocessing pipeline, and no preprocessing to a large clinical dMRI dataset (N = 524). Then, to assess the accuracy and precision of these new techniques, we created ground truths and induced Gibbs ringing or added noise for evaluation.

The outline of this paper is as follows: We first describe the new correction options, and then show comparison of the effect of DESIGNER-v2 pipeline against a minimal preprocessing pipeline and no preprocessing on conventional dMRI parameter estimation. We compare pipelines of different complexities by looking at age correlation after preprocessing with the pipelines in white matter of controls, an extensively studied topic (Beck et al., 2021; Kodiweera et al., 2016; Maximov et al., 2019; Ouyang et al., 2021; Schilling et al., 2022; Taha et al., 2022; Toschi et al., 2020; Yeatman et al., 2014). In addition, we evaluated the accuracy and precision of the modified noise removal and Gibbs correction steps through ground truth comparison. We also assessed the added effect of Gaussian smoothing in the minimal preprocessing pipeline in comparison to applying noise removal. For this study, we focused on evaluating the artifact correction steps only, hence excluding any steps beyond this, such as outlier detection (Andersson et al., 2016; Chang et al., 2012), smoothing, and tensor estimation methods (Collier et al., 2015; Tristán-Vega et al., 2012; Veraart et al., 2013). This study introduces a more robust and accurate DESIGNER-v2 pipeline as an optimized preprocessing pipeline for conventional multi-shell dMRI as acquired in clinical (research) settings.

## 2 Methods

### 2.1 Clinical data

We retrospectively studied control subjects (N = 524, 363 females, 161 males) with ages ranging from 25- to 75-year-olds. They were selected out of 5399 subjects who came in for routine clinical brain MRI on a Magnetom Prisma 3 T (N = 262) or Skyra 3 T (N = 262) (Chen et al., 2023). Control subjects were identified by clinical indication of dizziness or headache without MRI abnormalities and without history of neurological disease upon chart reviews (Pang et al., 2022; Liao et al., 2024). This retrospective study was reviewed and approved by the Institutional Review Board (IRB) in the United States (IRB number: i14-01224). Waiver of consent was obtained.

dMRI was acquired with the following protocol: 5 *b* = 0 images, 1 *b* = 0 image was acquired with reverse phase-encoding direction for EPI distortion correction (Andersson et al., 2003; Smith et al., 2004), *b* = 250 s/mm^2^ – 4 directions, *b* = 1000 s/mm^2^ – 20 directions, *b* = 2000 s/mm^2^ – 60 directions, TE = 70 ms (N = 142) or 95 ms (N = 382), TR = 3.7 s, 50 slices, resolution = 1.7 x 1.7 x 3 mm^3^, 6/8 partial Fourier, GRAPPA acceleration 2.

This clinical dMRI protocol included acquiring clinical diffusion tensor imaging (DTI) series (*b* = 0, 1000 s/mm^2^) alongside a research high *b*-value series (*b* = 0, 250, 2000 s/mm^2^) with the same imaging parameters (including TE) in both series. The high *b*-value series allows us to be sensitive to non-gaussian diffusion (Jensen & Helpern, 2010) and extract diffusion kurtosis imaging (DKI) metrics. However, high *b*-values require strong gradients and longer TE (applied to all *b*-values), which leads to increased distortion, lower signal-to-noise ratio (SNR), and longer scan time (Dietrich et al., 2001; Jones & Cercignani, 2010). Also, as a two series protocol, there may be intensity variations between the two series. Furthermore, this dMRI acquisition scheme came with several potential artifacts, including Gibbs ringing from partial Fourier and EPI distortion, making this an ideal dataset to test preprocessing pipelines (Chen et al., 2023).

### 2.2 Simulation I- HCP noise phantom

HCP phantom was generated as described by Ades-Aron et al. (2018). The HCP phantom was then modified by projecting spherical harmonics along directions matching the clinical data described above so it can mimic the clinical data. Next, it was downsampled to 2.5 x 2.5 x 2.5 mm^3^ (72 slices) and used as ground truth. Then, complex Gaussian noise was added using the equation $S_{m}=\sqrt{{\left(S_{r}+\sigma \text{​}\in_{1}\right)}^{2}+{\left(\sigma \text{​}\in_{2}\right)}^{2}}$ where $S_{m}$ is the simulated Rician-distributed magnitude MRI signal, $S_{r}$ is the real-valued signal, σ is the noise level, and $\in_{1}$ and $\in_{2}$ are independent normal variables with zero mean and unit variance, to create 50 sets of HCP phantom with SNR of 10, 15, 20, 25, 30, and 60. Note, without loss of generality, the imaginary signal component was set to be zero. Phantoms with varying SNR were generated to show the denoising effect on varying degrees of noise.

### 2.3 Simulation II- Shepp-Logan phantom for Gibbs ringing evaluation

Numerical simulation of the 2D Shepp-Logan phantom, a mathematical model of a brain made up of ellipses with varying size and signal intensities (Jain, 1989; Shepp & Logan, 1974), was used to assess Gibbs ringing removal methods. As the HCP phantom was originally created from dMRI datasets acquired with partial Fourier, it could not be used to evaluate partial Fourier-induced Gibbs ringing removal methods because it had remaining Gibbs ringing from partial Fourier. Instead, Gibbs ringing from k-space truncation and 6/8 partial Fourier in the horizontal direction was introduced to Shepp-Logan phantom.

### 2.4 Experiments

#### 2.4.1 Preprocessing pipelines

**Fig. 1.**
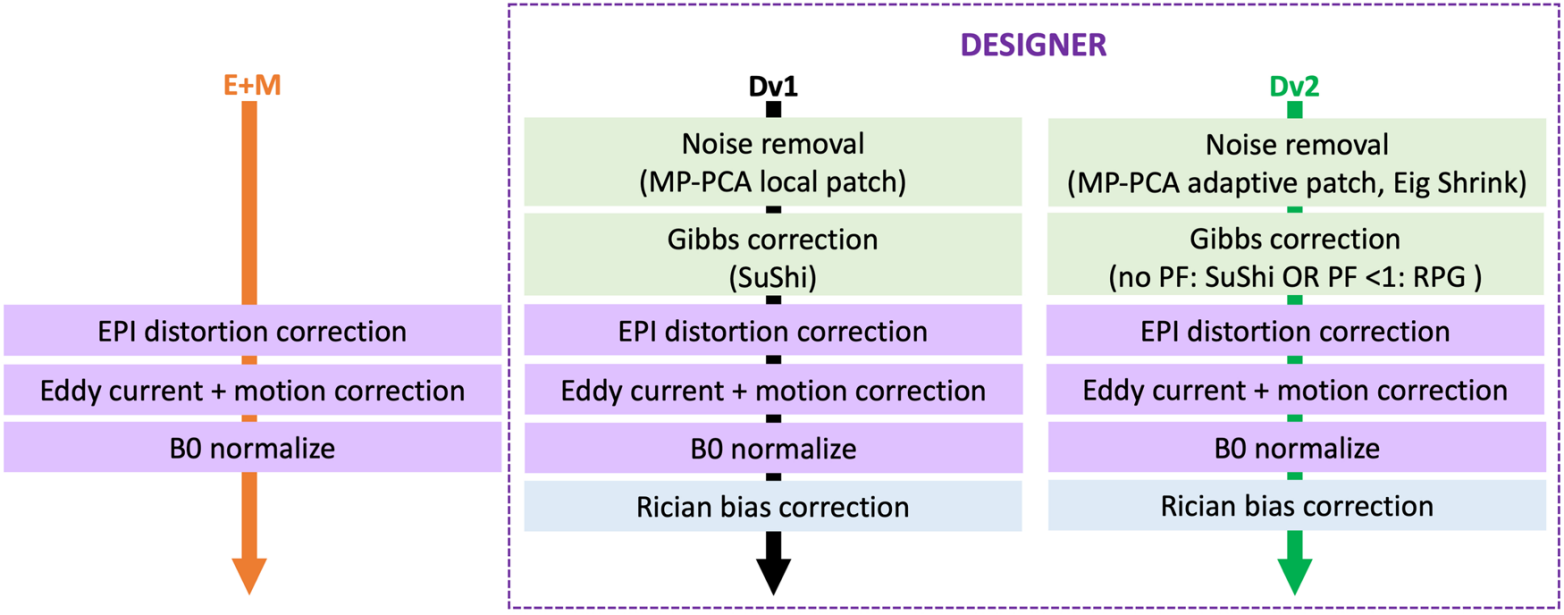
Step-by-step flow diagram for E+M, original DESIGNER-v1 (Dv1), and current DESIGNER-v2 (Dv2). Eig Shrink: eigenvalue shrinkage, PF: Partial Fourier, SuShi: Subvoxel-Shifts, RPG: Removal of partial Fourier-induced Gibbs.

Figure 1 shows the flow diagram of the main pipelines employed on clinical data in this study. Below lists all pipelines applied on clinical data:

1. The minimal preprocessing pipeline targets EPI distortion (Andersson et al., 2003), eddy current (Andersson & Sotiropoulos, 2016), and motion correction (Andersson et al., 2016). We call this Eddy/topup+Motion (E+M) pipeline. Since the clinical dataset included two series, E+M also includes b0 normalization, which does voxelwise rescaling by taking the ratio of smoothed (Gaussian kernel (mm) with 3 standard deviations) *b* = 0 images from each series to rescale all diffusion-weighted images. This pipeline was based on the routinely used HCP diffusion preprocessing pipeline (Glasser et al., 2013).
2. Original DESIGNER-v1 (Dv1) is a previously validated (Ades-Aron et al., 2018) pipeline involving the same corrections as the E+M pipeline defined above. It additionally deals with thermal noise, Gibb ringing, and Rician signal biases using local patch MPPCA denoising (Cordero-Grande et al., 2019; Veraart, Novikov, et al., 2016), subvoxel-shifts (SuShi) Gibbs removal (Kellner et al., 2016), and Rician bias correction (Koay & Basser, 2006), respectively.
3. DESIGNER-v2 (Dv2) is an updated version of DESIGNER-v1. Recent technical developments promote a more efficient denoising and wider applicable Gibbs removal method, as outlined by the following updates:
  - Adaptive patch MPPCA denoising with eigenvalue shrinkage (Gavish & Donoho, 2017) in Dv2 creates patches of voxels from the same tissue type to maximize signal redundancy during denoising and, as such, the denoising performance. The adaptive patch selection uses a bilateral approach to select 100 voxels with the most similar underlying tissue content. For the central voxel in the patch, we choose voxels with both small Euclidean distance to this voxel and voxels with the smallest difference in intensity (over all diffusion directions).
  - Removal of partial Fourier-induced Gibbs ringing (RPG) (Lee et al., 2021) was developed to target ringing from both symmetric and asymmetric k-space truncation. Partial Fourier acquisition is common in clinical (research) settings and leads to both types of ringing. While SuShi (Kellner et al., 2016) Gibbs removal works on ringing from symmetric k-space truncation, asymmetric k-space truncation ringing pattern remains. This has led to the development of RPG (Lee et al., 2021), which additionally removes ringing from asymmetric k-space by resampling the image so SuShi Gibbs removal targets this remaining ringing on the resampled image. RPG works best on data with 6/8 and 7/8 partial Fourier.
4. To compare the Dv2 pipeline with one that simply uses smoothing to deal with noise, we applied E+M pipeline with minimal Gaussian smoothing (full width at half maximum (FWHM) of 1.2*voxel size) to the clinical dMRI.
5. To study the effect of adaptive patch MPPCA denoising with eigenvalue shrinkage (including Rician bias correction) separately from RPG, Dv2, as described above, but without Gibbs correction was also applied to clinical dMRI.
6. To study the effect of RPG separately from denoising and Rician bias correction, Dv2 without denoising and Rician bias correction was applied to the clinical dMRI.

E+M, Dv1, and Dv2 pipelines were applied using options within DESIGNER available for installation at https://github.com/NYU-DiffusionMRI/DESIGNER-v2.

#### 2.4.2 Clinical dMRI experiments

Using the clinical dMRI scans, we tested how applying pipelines with different corrections affect parameter outcomes using a minimal preprocessing pipeline versus a more advanced preprocessing pipeline involving noise removal and Gibbs ringing removal. DTI (MD – mean diffusivity (µm^2^/ms), RD – radial diffusivity (µm^2^/ms), AD – axial diffusivity (µm^2^/ms), and FA – fractional anisotropy) and DKI (MK – mean kurtosis, RK – radial kurtosis, AK – axial kurtosis) parameter maps from clinical dMRI were extracted using weighted linear least squares fit (Veraart et al., 2013) after preprocessing the DWIs (diffusion-weighted images) in three different ways: (1) no artifact or noise removal, (2) E+M, and (3) Dv2. Then, they were compared visually and quantitatively. Clinical data were also preprocessed with Dv1 and compared to Dv2.

**Fig. 2.**
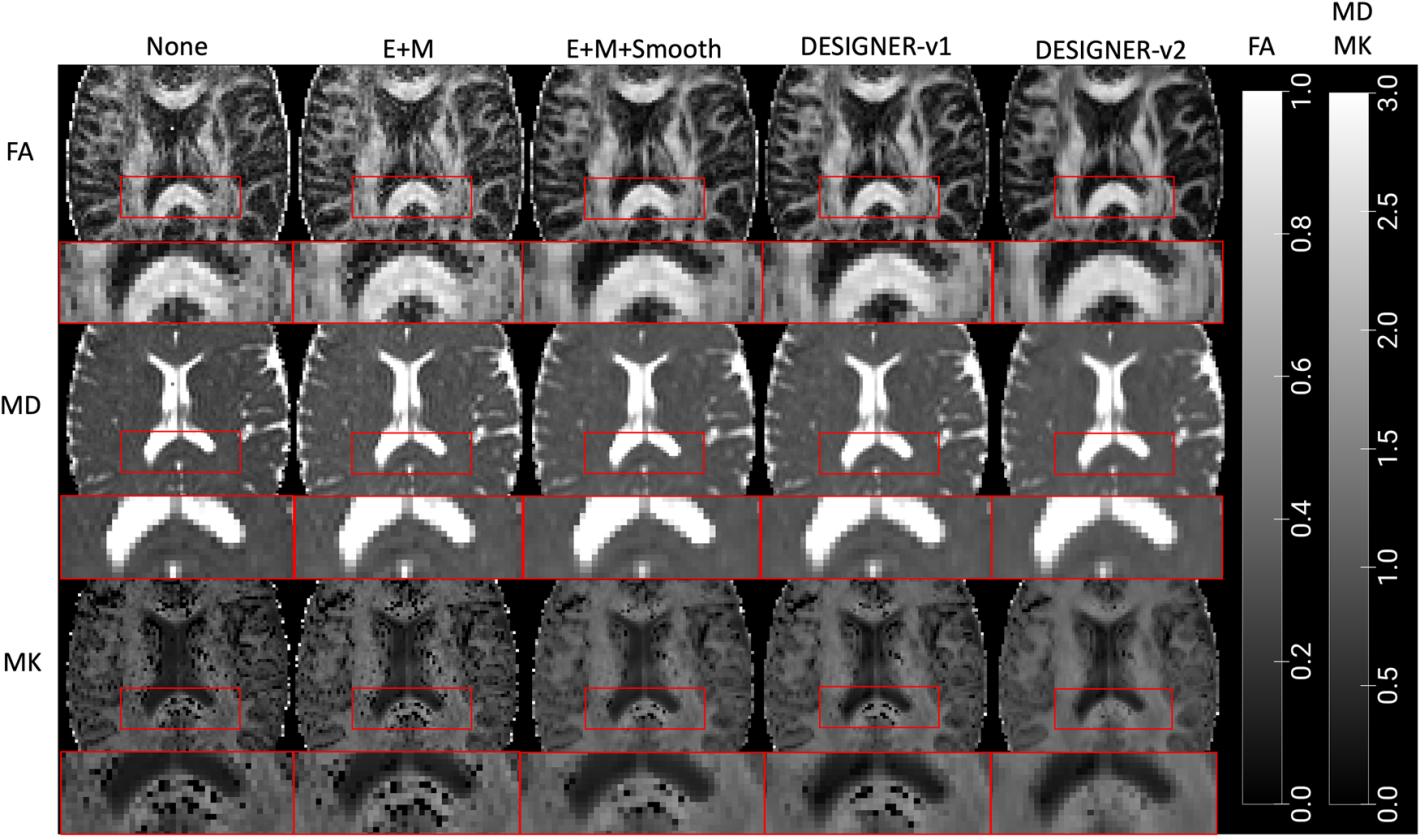
Parameter maps (FA, MD, MK) of a healthy 60-year-old female based on preprocessing of dMRI in five different ways: (1) no preprocessing, (2) E+M, (3) E+M+Smooth (CSF excluded Gaussian smoothing with FWHM = 1.2*voxel size), (4) DESIGNER-v1, and (5) DESIGNER-v2 pipeline. DESIGNER-v2 results in parameter maps with the least noise and outliers, and mostly reduced Gibbs ringing in the splenium/corpus callosum boundary (bright band in FA, dark band in MD, and black voxels in MK).

To look at the effect of smoothing to deal with noise, clinical dMRI scans were also preprocessed with E+M pipeline in addition to CSF excluded Gaussian smoothing with FWHM of 1.2*voxel size (E+M+Smooth). Parameter maps were then compared for those without preprocessing, E+M, E+M+Smooth, Dv1, and Dv2 pipelines applied (Fig. 2).

Outlier voxels across all DTI/DKI parametric maps were defined based on their physically possible lower and upper bounds: diffusivity D (MD, AD, RD) > 0, fractional anisotropy 0 < FA < 1, and kurtosis (Dubkov & Malakhov, 1976) K (MK, AK, RK) > -2. Outliers are values outside of these bounds (the upper bound of D < 3 is rarely violated, so it was not included here). We calculated average outlier percentage across parameter maps over each region of interest (ROI) (described below) to quantify remaining outlier voxels after preprocessing with each pipeline.

**Fig. 3.**
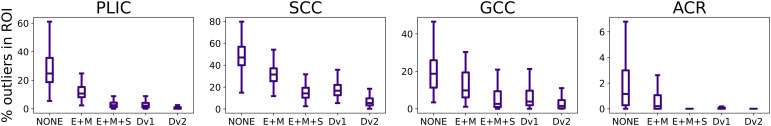
Boxplots of percent outliers (100*number of outliers in ROI/number of voxels in ROI) in each ROI of the dMRI parameter maps of 524 subjects show outliers are reduced from no preprocessing to E+M, E+M+Smooth, DESIGNER-v1, and DESIGNER-v2 preprocessing pipeline.

For quantitative measures, to exclude boundary voxels affected by partial volume effect, JHU white matter atlas ROIs (Hua et al., 2008) were each clipped in atlas space to exclude 5% of the lowest FA. They were then warped to each participant’s FA map by first applying a linear registration using FSL’s FLIRT (FMRIB’s Linear Image Registration Tool) (Jenkinson et al., 2002; Jenkinson & Smith, 2001) which was used to initialize the nonlinear warp using FNIRT (Andersson et al., 2010). For studying the effect of the pipeline on age correlations, we extracted the median from each parameter map for ROIs of the posterior limb of internal capsule (PLIC), splenium (SCC), genu of corpus callosum (GCC), and anterior corona radiata (ACR). The median was selected to reduce the effect of outliers in the ROIs, which varies between pipelines (Fig. 3). These regional metrics were then plotted with respect to age. Note that our clinical dMRI contain images acquired with either TE = 70 ms or TE = 95 ms. To avoid confounding effects due to mixing TEs, as shown in figure S1, and scanners, age correlations were plotted for data acquired by the same TE and scanner (Prisma, Skyra).

A quadratic fit was plotted to show age association as adjusted R^2^ values using quadratic fit is greater when compared to adjusted R^2^ using linear fit for all age associations (Table S1). Bonferroni-adjusted P-values (multiply p-value by 28 (7 parameters x 4 ROIs)), concavity, and age at inflection point (start of decline) were collected from each parameter-ROI pairing to compare the effect pipelines have on age association. Significance level was set to Bonferroni adjusted P-values < 0.05. Since the age range is 25 to 75 years old, the quadratic fit for that range may not include an extremum. In that case, instead of indicating concavity and age at inflection point, we indicated if DTI/DKI parameters are monotonically increasing or decreasing.

Additionally, we considered the effects of denoising and Gibbs ringing removal separately on the clinical dMRI, by preprocessing with pipelines (5) and (6) described above. Percent differences from E+M derived metrics were quantified for each scan in SCC, GCC, ACR, and PLIC ROIs (mean of ROI after omitting outliers).

#### 2.4.3 Simulation experiments

To evaluate the updated denoising method in Dv2, various denoising methods were applied to the HCP noise phantoms. Phantoms were only preprocessed with noise removal and/or Rician bias correction as we only introduced complex Gaussian noise to the ground truth and we were only interested in comparing the denoising methods here. They were preprocessed using (1) E+M’s (no denoising), (2) Dv1’s (local patch denoising followed by Rician bias correction), and (3) Dv2’s (adaptive patch denoising with eigenvalue shrinkage followed by Rician bias correction) denoising method. Noisy phantoms were also preprocessed with (4) adaptive patch denoising with eigenvalue shrinkage (without Rician bias correction) and (5) adaptive patch denoising without eigenvalue shrinkage followed by Rician bias correction to compare effect of Rician bias correction and eigenvalue shrinkage separately.

For each parameter, maps were computed as the median over 50 noise realizations for a given SNR level. We took the median rather than the mean to avoid maps resulting in only outliers for low SNR phantoms. Denoising methods were evaluated by calculating, for each denoising method, a median percentage error map against their respective ground truth parameter maps. Similar to clinical data, JHU white matter atlas ROIs were warped to HCP phantom’s ground truth FA map, but then merged into an overall WM ROI. We then extracted and plotted the median over the WM ROI from the median percentage error map to compare percent error after applying the different denoising methods. Additionally, average outlier percentage across parameter maps over WM ROI was calculated to quantify remaining outlier voxels, defined as D < 0, K < -2 or FA > 1, after each denoising method.

To evaluate smoothing to deal with noise, CSF excluded Gaussian smoothing with full width at half maximum (FWHM) of 1.2*voxel size) was applied on a set of HCP noise phantoms (SNR 10, 15, 20, 25, 30, 60). Then, we extracted median percent error in SCC from parameter maps yielded from: (1) no noise removal (2) Gaussian smoothing, (3) adaptive patch denoising with eigenvalue shrinkage followed by Rician bias correction, and (4) adaptive patch denoising without eigenvalue shrinkage followed by Rician bias correction.

The Gibbs Shepp-Logan phantom was preprocessed with E+M’s (no Gibbs removal), Dv1’s (Subvoxel-shifts Gibbs removal), or Dv2’s (RPG) Gibbs correction method to evaluate the updated Gibbs removal method in Dv2. Only Gibbs removal was used on the phantom since we are only evaluating the Gibbs removal methods here and no other image artifact was introduced to the phantom. Gibbs ringing removal methods were assessed by generating mean percentage error against ground truth phantom over four manually drawn ROIs targeting Gibbs ringing artifacts. Smoothing effects from applying weighting filters and simulation with full-Fourier acquisition have been evaluated previously by (Lee et al., 2021), and thus not explored here.

## 3 Results

### 3.1 Clinical MRI

#### 3.1.1 Effect of preprocessing pipelines on age association of diffusion parameters in white matter

Figure 2 shows FA, MD, and MK maps of a 60-year-old female are the least noisy after preprocessing with DESIGNER-v2 as compared to no preprocessing, E+M, E+M+Smooth, or DESIGNER-v1. In the SCC/CSF boundary, the Gibbs artifact in SCC/CSF boundary (bright band in FA, dark band in MD, and black voxels in MK) was greatly reduced after preprocessing with RPG from Dv2. In addition, the MK maps show improvement as the number of negative outliers or black voxels drastically decrease with E+M+Smooth, Dv1, and Dv2 when compared to no preprocessing and E+M.

The effect of preprocessing on the presence of outliers in parametric maps of all 524 clinical dMRI is shown in Figure 3, plotting the percent outliers in PLIC, SCC, GCC, and ACR after no preprocessing, E+M, E+M+Smooth, Dv1, and Dv2 preprocessing. SCC generally has the most outliers, followed by PLIC, GCC, and finally ACR. Results from Tukey’s HSD test for multiple comparisons show percent outliers in each of the four ROIs were significantly different between all pipelines (p-value < 0.05) except between Dv1 and Dv2 in PLIC, E+M+Smooth and Dv1 in PLIC, SCC, and GCC, E+M+Smooth and Dv2 in GCC, and between E+M, E+M+Smooth, Dv1, and Dv2 in ACR. Employing more advanced pipelines decreased the percentage of outliers in ROIs. The systematic reduction in outliers after E+M+Smooth, Dv1, and Dv2 preprocessing is in line with what we see in Figure 2.

**Fig. 4.**
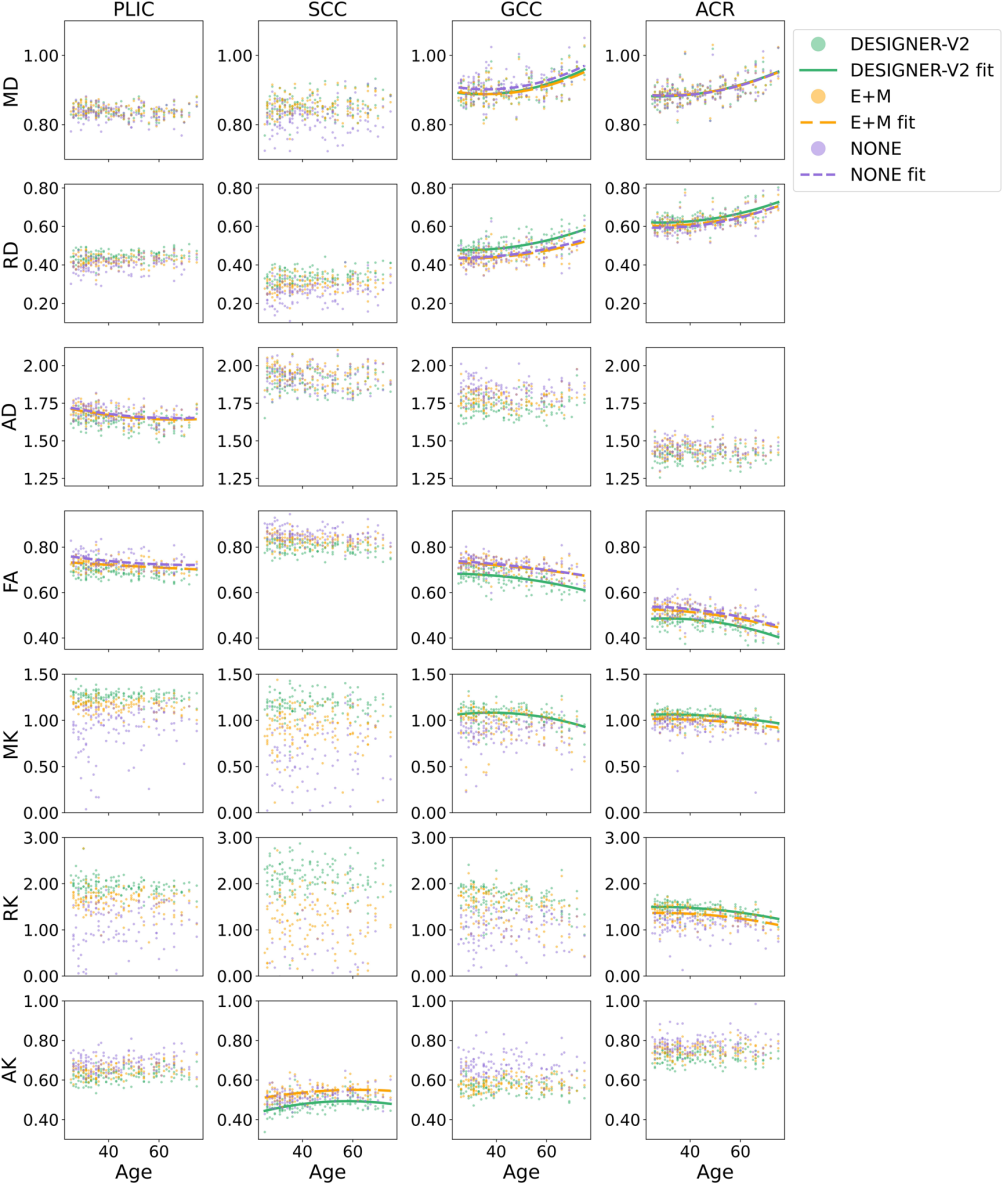
Age correlation with DTI and DKI parameters in white matter ROIs (median) derived from DESIGNER-v2 pipeline, E+M, or no preprocessing of 142 healthy subjects (Prisma, TE = 70 ms). Quadratic fits were plotted for statistically significant correlations with adjusted R^2^ > 0.1 only (Tables S1-S2). MK and RK plots are zoomed in so some outlier datapoints (Fig. 3) are not shown.

Figure 4 shows age correlations of DTI/DKI parameters of 142 control subjects (TE = 70 ms, Prisma) derived from Dv2, E+M, and no preprocessing (none) in ACR, PLIC, GCC, and SCC. For all ROIs and parameters, the observed age dependencies were similar in shape for the curves obtained with different preprocessing pipelines. However, as also observed in histograms in Figure S3, the type of preprocessing resulted in some systematic differences in absolute parameter values: compared to no preprocessing and E+M, when preprocessed with Dv2 pipeline, (1) FA and AK were systematically lower while (2) RD, MK, and RK were systematically higher. Preprocessing pipelines also had varying impact in different metrics. For example, Figure 4 reveals that preprocessing pipelines did not affect MD as much as other metrics in all ROIs. Similar results are observed in Figures S7 and S9 for data acquired with TE = 95 ms.

The strength of the observed age associations also depended on the preprocessing pipeline. In Table S1, adjusted R^2^ in ACR and GCC consistently increases from no preprocessing to Dv2 for MD, RD, RK, MK, and FA. Correlations either increased or decreased in strength with different pipelines (Table S2). For example, for MK and RK in ACR and GCC, from no preprocessing to Dv2, correlations increased as a more advanced preprocessing pipeline was applied. On the other hand, correlations decreased in strength for the AD in all ROIs from no preprocessing to Dv2. Age correlation results for the remaining data (TE = 95 ms for Prisma and Skyra) are shown in Tables S3-S6. Overall, p-values and adjusted R^2^s from TE = 95 ms data agreed with results from TE = 70 ms data (Tables S1-S2).

Figures S2, S4, S6, S8, and S10 compare Dv1 and Dv2. Overall, age association adjusted R^2^ and p-values (Tables S2-S2) were similar between the DESIGNER pipelines. Some systematic differences in absolute parameter values were observed in Figures S2 (Prisma, TE = 70 ms), S8 (Prisma, TE = 95 ms), and S10 (Skyra, TE = 95 ms), which all showed that compared to Dv1, preprocessing with Dv2 had systematically (1) higher RD and MK and (2) lower FA. Additionally, Figure S6 compares coefficient of variation defined by standard deviation/mean of ROI and ROI coefficient of variation from Dv2 was consistently lower than Dv1 and other pipelines (Fig. S5).

Table 1 summarizes concavity, age at inflection point, and if parameters were monotonically increasing or decreasing from 25 to 75 years old for statistically significant age associations with adjusted R^2^ > 0.1. Based on the type of preprocessing pipeline, there was disagreement on whether the quadratic fit was in the concave portion of the quadratic model or monotonically increasing or decreasing for FA in PLIC and ACR.

**Table 1:**
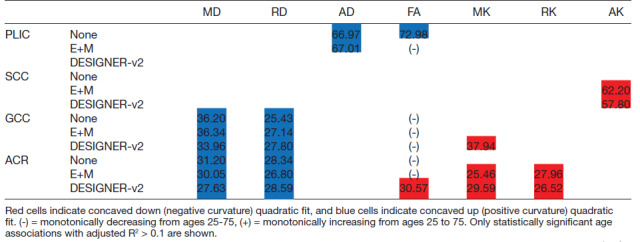
Age at peak (in years) from DTI and DKI age correlation quadratic fits in white matter regions using no preprocessing, E+M, and DESIGNER-v2 preprocessing pipeline.

#### 3.1.2 Effect of updated denoising versus Gibbs correction

**Fig. 5.**
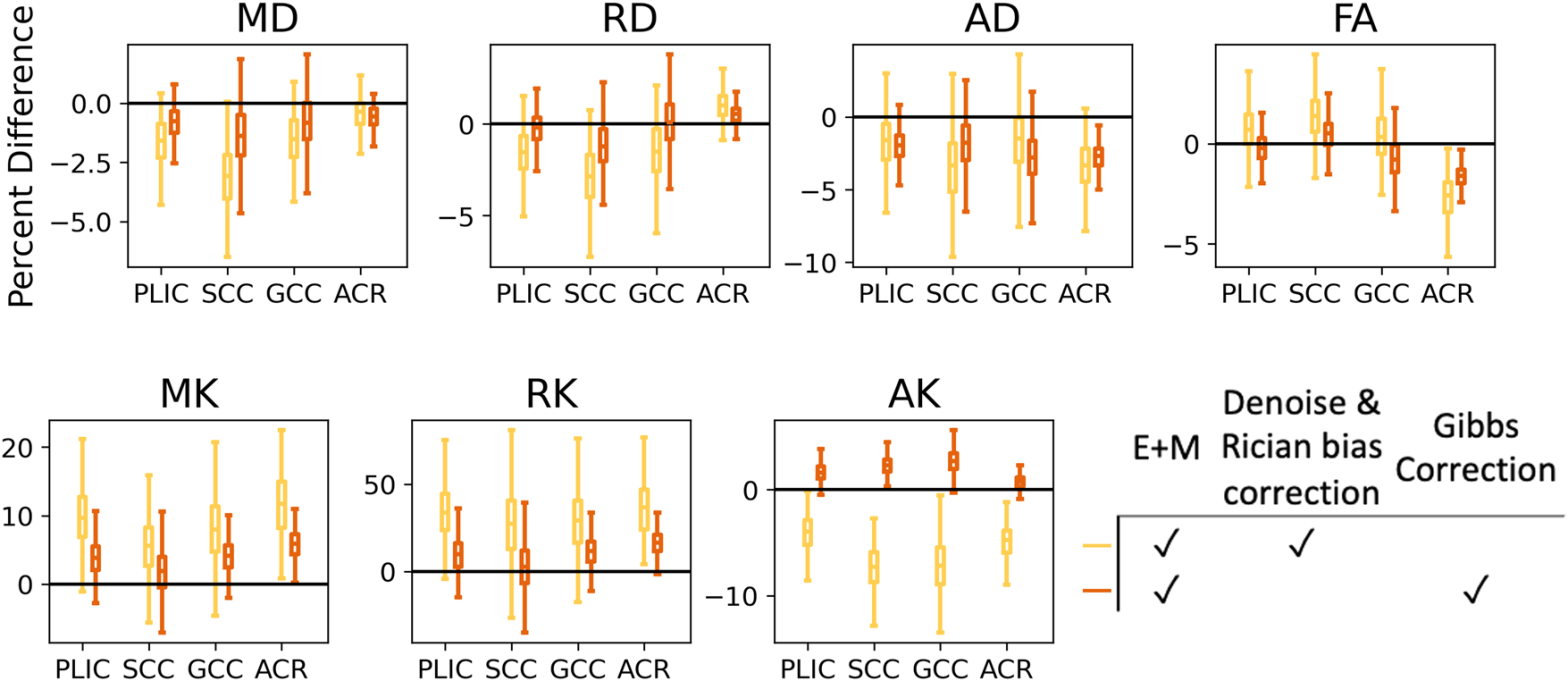
Boxplots of mean percent difference from E+M preprocessing after denoising with adaptive patch with eigenvalue shrinkage (yellow) or Gibbs ringing removal with RPG (red) for 524 healthy subjects on ROI level (mean after omitting outliers). One-way ANOVA showed the effect of adaptive patch denoising with eigenvalue shrinkage versus RPG was significantly different for all metrics in each ROI (p-value < 0.05) except MD in ACR and AD in PLIC and GCC.

The effects of DESIGNER-v2’s adaptive patch denoising with eigenvalue shrinkage (and Rician bias correction) and RPG in clinical data are quantified separately with respect to E+M in Figure 5 as box plots. Results from one-way ANOVA show that the effect of adaptive patch denoising with eigenvalue shrinkage versus RPG was significantly different for all metrics in each ROI (p-value < 0.05) except MD in ACR and AD in PLIC and GCC. The boxplots reveal denoise and bias correction can have opposite effects from Gibbs correction (AK in all ROIs, FA in GCC). There was also generally a greater effect from adaptive patch denoising with eigenvalue shrinkage than from RPG. Additionally, DESIGNER-v2’s denoise and Gibbs removal steps affected MK and RK in each ROI more than DTI metrics.

#### 3.1.3 Preprocessing time

The preprocessing time for each clinical dataset did not differ much from pipeline to pipeline. The most time-consuming step was eddy current and motion correction. On a 24 CPU core Ubuntu Linux server with 256 GB memory, E+M completed in approximately 45 minutes while noise and Gibbs removal from DESIGNER-v1 and DESIGNER-v2 took up an additional 5 and 7 minutes to complete respectively.

### 3.2 Simulations

#### 3.2.1 Assessment of denoising methods on HCP phantom

**Fig. 6.**
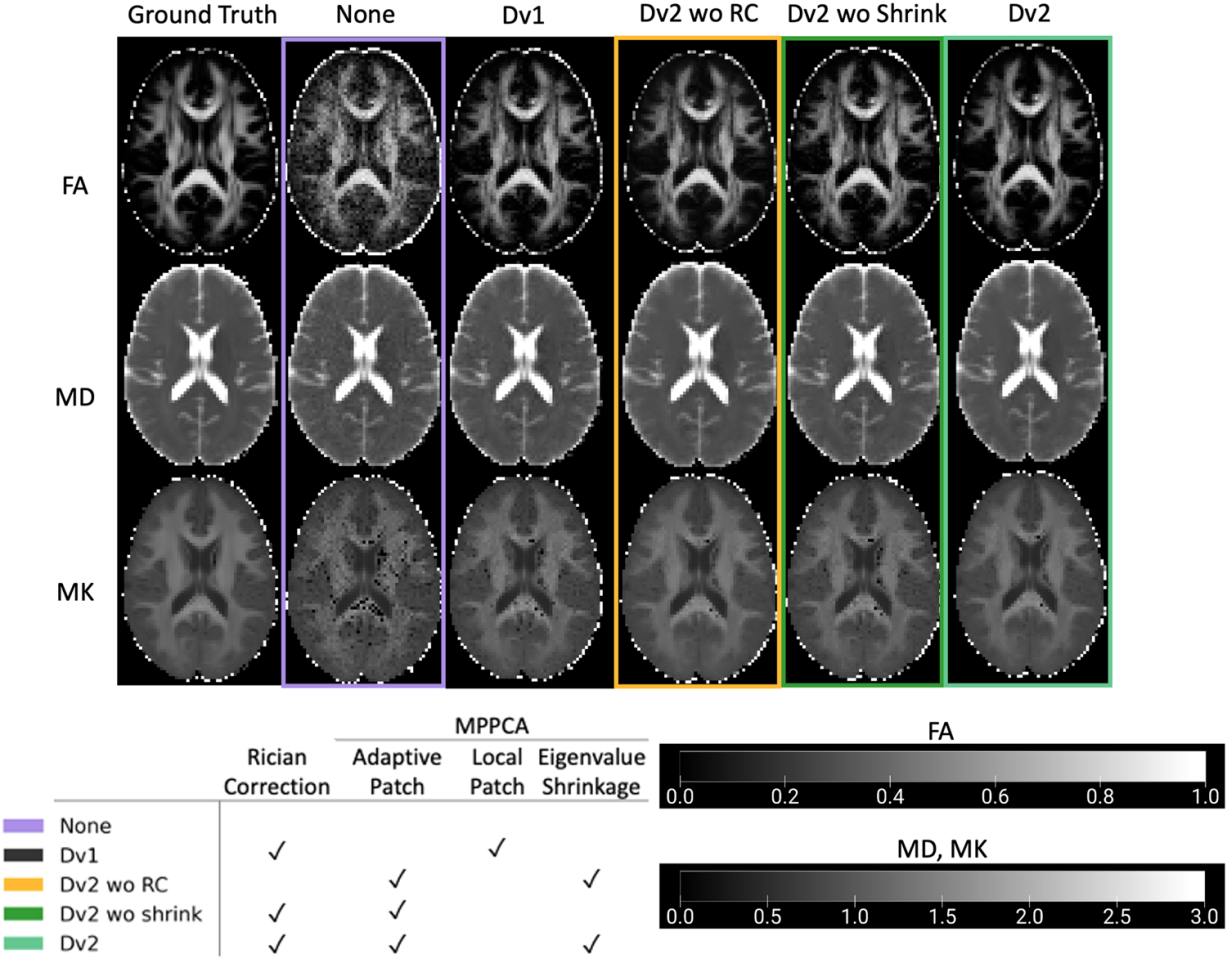
FA, MD, and MK maps for ground truth HCP phantom, noisy phantom (SNR 20), and after varying denoising methods. Maps with adaptive patch denoising with eigenvalue shrinkage applied appears to have the least black voxels.

Figure 6 shows FA, MD, and MK maps of HCP noise phantom (SNR = 20) with varying denoising methods applied. We observed reduced outliers due to noise when processing with denoising methods from Dv1 and Dv2 versus no denoising (None). We also observed adaptive patch (Dv2) slightly improved denoising performance over local-patch (Dv1). Furthermore, eigenvalue shrinkage (Dv2) appeared to have an additional denoising effect, yielding less noise and outlier voxels.

**Fig. 7.**
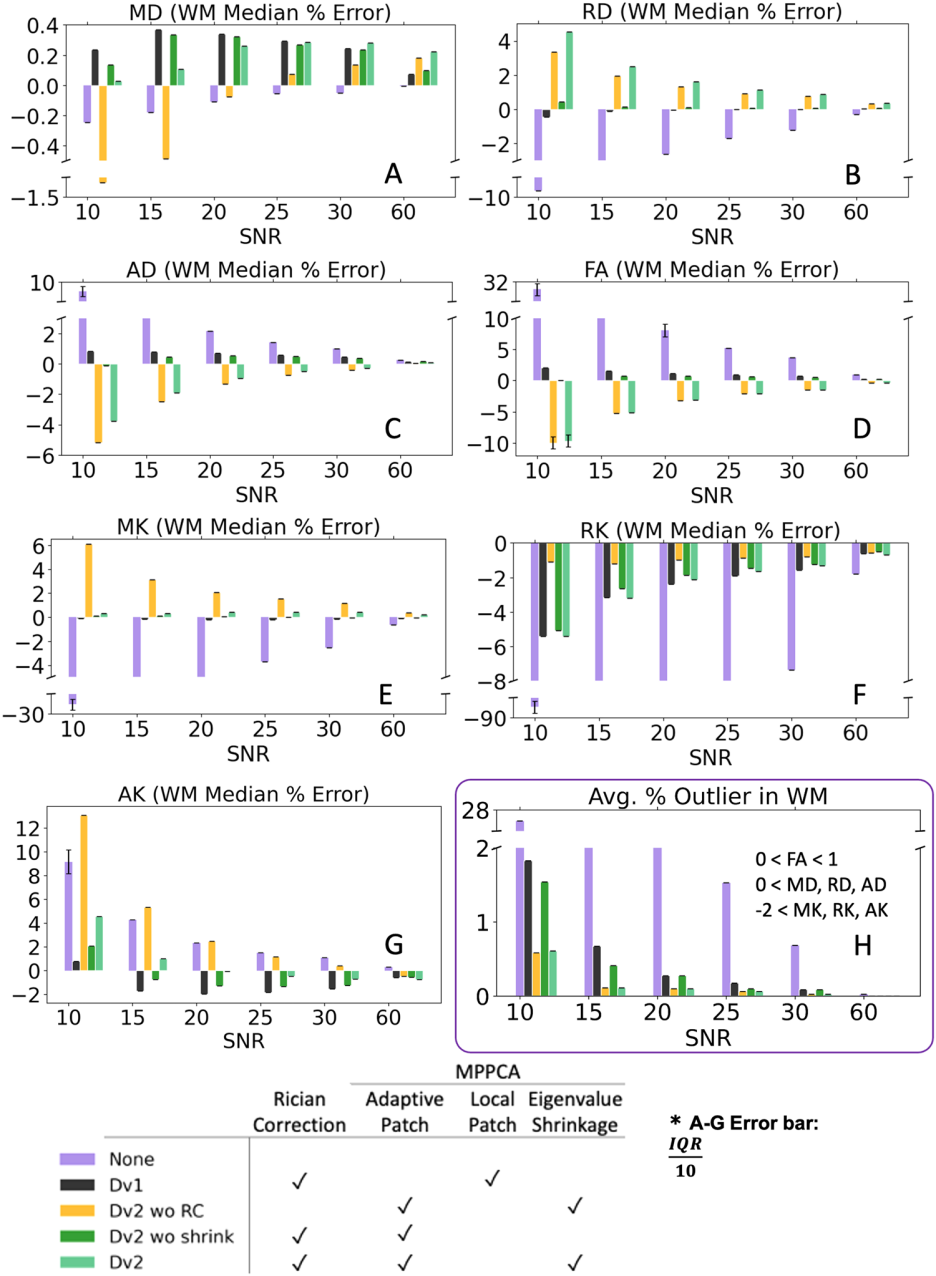
Ground truth evaluation of noise removal methods. **(**A-G) Bar plots showing median and interquartile range (error bar scaled by 1/10) of DTI/DKI parameters median percentage error in WM of 50 sets of phantoms (SNR from 10-60). (H) Average percent outlier (WM) of 50 phantom iterations (SNR from 10-60). Note for SNR between 30 and 60, denoising became obsolete in MD (where median percent error is only within 1.5% across all SNR even without any correction) and AK.

Figure 7A-G plots medians over the WM ROI from median percentage error maps. The plots show no denoising resulted in the most bias in all parameters except AK and denoising without Rician bias correction had the most bias in AK. Overall, denoising with an adaptive patch without eigenvalue shrinkage yielded the most accurate result. Interestingly, denoising with eigenvalue shrinkage generally had some bias in the opposite direction of the noise bias in MD, RD, AD, and FA maps. This bias from eigenvalue shrinkage was opposite to the effect of eigenvalue repulsion. For example, with eigenvalue shrinkage, FA and AD had negative bias and RD had positive bias. However, both Figures 6 and 7H show kurtosis maps with eigenvalue shrinkage had the fewest outlier voxels. Outlier percentage in WM decreased from 0.27% to 0.10% when using adaptive patch followed by Rician bias correction versus adaptive patch with eigenvalue shrinkage followed by Rician bias correction respectively on noisy phantom with SNR = 20. Eigenvalue shrinkage appeared to over-correct for noise (more bias with more noise) to give a smoothing effect on the parameter maps.

**Fig. 8.**
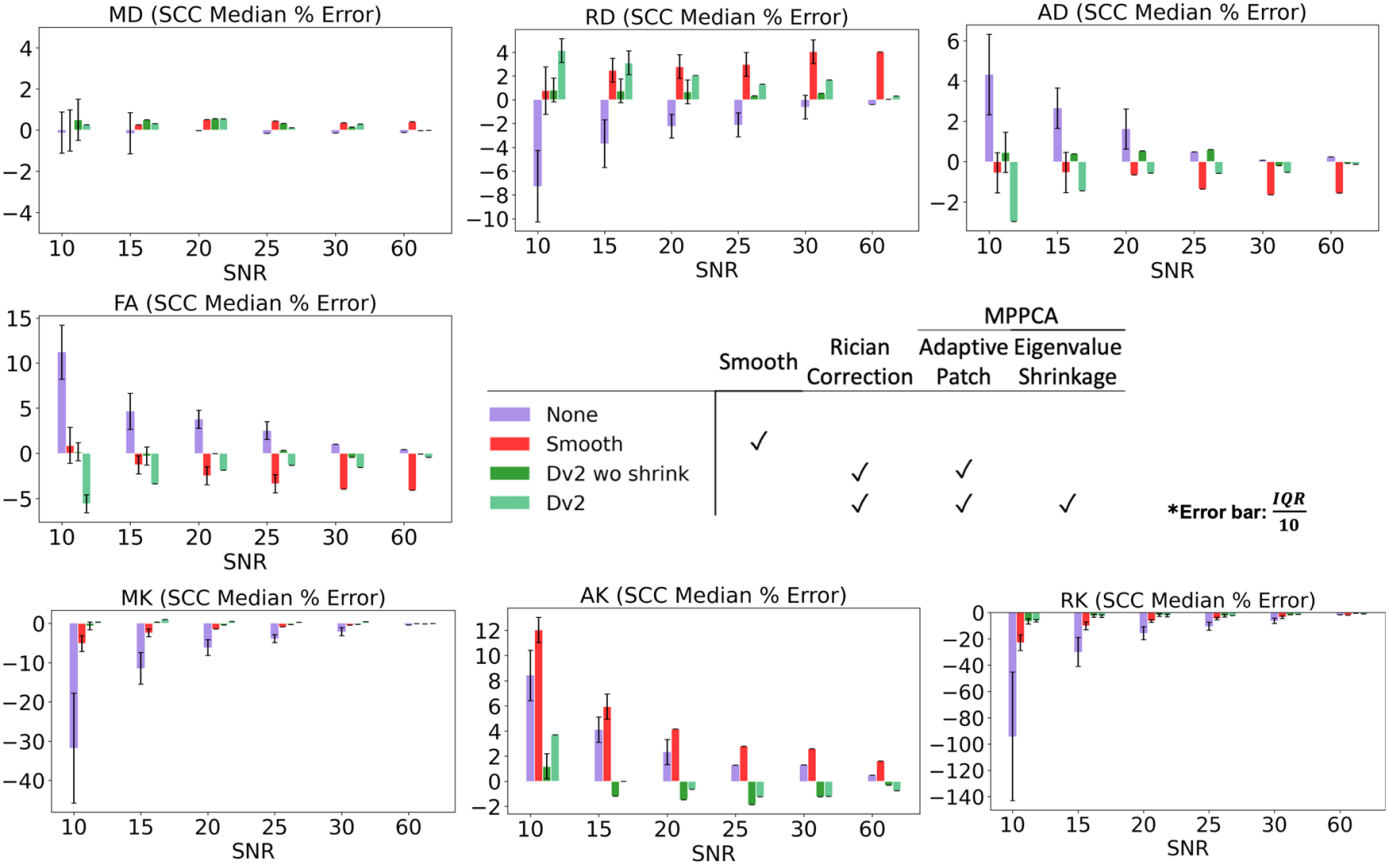
Ground truth evaluation of CSF excluded Gaussian smoothing and noise removal methods show minimal smoothing helps reduce noise at SNR<20, but is harmful at SNR>20 and does not perform as well as denoising using Dv2 preprocessing. Bar plots show median percentage error and interquartile range (error bar scaled by 1/10) of DTI/DKI parameters in SCC of a set of noise phantoms (SNR from 10-60).

Figure 8 compares median percent error from ground truth in SCC after smoothing and denoising a set of HCP noise phantoms. At low SNR of 10-20, smoothing improved accuracy in all metrics except AK. However, at SNR of 25-60, smoothing worsened accuracy in all metrics besides MK and RK. Except at low SNR of 10-20 in MD, adaptive patch denoising with eigenvalue shrinkage followed by Rician bias correction (Dv2) and/or adaptive patch denoising without eigenvalue shrinkage followed by Rician bias correction (Dv2 wo shrink) performed better than smoothing.

#### 3.2.2 Assessment of Gibbs ringing correction on Shepp-Logan phantom

**Fig. 9.**
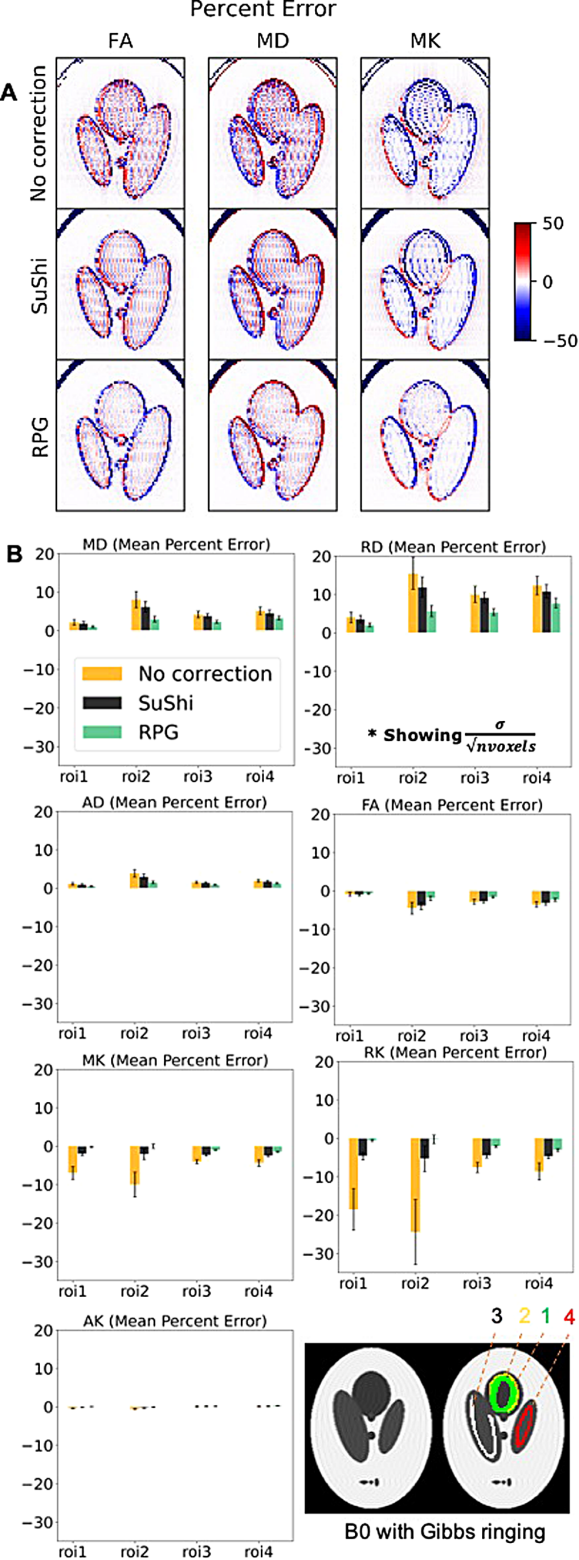
Evaluation of Gibbs removal. DTI/DKI mean percentage error compared between Shepp-Logan phantom with Gibbs ringing (simulated from k-space truncation and 6/8 PF in the horizontal direction) without correction, corrected with SuShi method, and corrected with RPG. (A) FA, MD, and MK mean percentage error maps. (B) Bar plots showing ROIs’ mean percentage error and standard error in DTI/DKI parameter maps.

Figure 9A shows DTI/DKI mean percentage error maps for no Gibbs correction and Gibbs correction using SuShi or RPG. The maps show that the SuShi Gibbs correction method removed some Gibbs ringing artifacts while using RPG removed additional ringing resulting from partial Fourier. Figure 9B shows mean percentage error in each manually drawn ROI targeting Gibbs ringing. Gibbs removal using RPG was the most accurate, followed by SuShi method, while not applying correction had the greatest percent error in all ROIs and parameters.

## 4 Discussion

This study reveals improved accuracy and robustness of DTI/DKI voxelwise estimation and thus more reliable results when multi-shell dMRI acquisitions are preprocessed with Dv2 pipeline compared to no preprocessing, E+M, and Dv1 pipeline. Our age correlation study displays how using different preprocessing pipelines induces variability in parameter values. Visualizing parameter maps help understand the importance of denoising and Gibbs removal for improving DTI and DKI maps. Additionally, ground truth results also show optimized correction methods from Dv2 yield more accurate and robust maps (Fig. S11, Fig. 9). The pipeline comparisons using clinical dMRI along with the simulated ground truth studies illustrate that using Dv2 pipeline yields more reliable diffusion parameter values.

To visualize the effect of noise and Gibbs removal, we looked at parameter maps from a healthy 60-year-old female. We confirmed reduced noise, Gibbs ringing, and outlier voxels in parameter maps as we introduced denoising and Gibbs ringing removal (Fig. 2). Considering all subjects, we also quantified percent outliers in ROIs and saw significant decrease in outliers as preprocessing steps were added (Fig. 3). Agreeing with Figure 2, in ROIs that are affected by Gibbs ringing such as corpus callosum, outliers were further reduced when targeting partial Fourier-induced Gibbs ringing in Dv2, which was not included in Dv1. While a previous study (Kornaropoulos et al., 2022) did not find improvement after denoising and Gibbs removal when performing analyses based on ROI-values, our results show that parametric maps from minimal processing (E+M) feature profoundly more outliers as compared to our DESIGNER pipelines due to denoising and Gibbs removal. These observations show we can expect improved voxelwise statistical results when appropriate correction strategies in dMRI data are used.

Age correlations (Fig. 4) revealed systematic differences between parameter values from varying pipelines in all ROIs for all metrics except MD. Additionally, it is known that late-myelinating tracts are more vulnerable to neurodegeneration (Bartzokis, 2004; Benitez et al., 2014). So, we expected changes with age due to demyelination reflected most in radial metrics (RD, RK) and FA in late-myelinating tracts (Bartzokis, 2004; Benitez et al., 2014; Paydar et al., 2014). Here (Table S1), as expected, adjusted R^2^ from no preprocessing to Dv2 is consistently increasing in the more vulnerable late-myelinating tracts (ACR and GCC) as compared to SCC and PLIC. Since myelin affects diffusion mostly in the radial direction, there is no clear biological effect due to demyelination in the axial metrics expected. Indeed, the low correlation in AD with no preprocessing is likely spurious as it further weakens with an improved pipeline, potentially due to improved artifact correction including Gibbs. Thus, preprocessing pipeline affects parameter values and statistical results. These findings highlight the importance of preprocessing all data with the same optimal pipeline and to be wary of mixing pipelines in studies.

To differentiate the effect of noise removal and the effect of Gibbs removal on DTI and DKI parameters from clinical dMRI, we used E+M as the baseline of comparison. The effect of Dv2’s noise removal and Gibbs removal are significantly different, and the corrections can have opposing changes as they are targeting different issues. Thus, for more reliable metrics, both denoising and Gibbs correction should be applied to address noise and Gibbs ringing. Additionally, Dv2’s denoising (adaptive patch denoising with eigenvalue shrinkage) and Rician bias correction generally contribute more to the change in resulting DTI and DKI ROI values than RPG does (Fig. 5). This is likely because Gibbs ringing appears as oscillations, so taking the mean percent difference over an ROI then cancels out the error. The systematic differences in absolute parameter values (Fig. 4) between E+M and Dv2 are mostly contributed to denoising and Rician bias correction.

Ground truth phantoms were used to evaluate the recent denoising method proposed in our Dv2 pipeline: MPPCA adaptive patch denoising (compared to local patch) with eigenvalue shrinkage and Rician bias correction. The absolute median percent error of DTI/DKI parameters in white matter was much greater when no noise removal was applied on phantoms with SNR up to 30 (Fig. 7), implying denoising at SNR of about 30 and below improves accuracy greatly. For SNR between 30 and 60, denoising became less beneficial (but also not harmful). While noise phantoms show denoising was the largest contributing factor to increase accuracy for most parameters, Rician bias correction contributed most to bias in AK.

The insights from phantom data help interpret the observed systematic differences in the clinical diffusion maps (Fig. 4) as being likely due to lowering noise floor in AK and reduction of eigenvalue repulsion from improved denoising in RD, FA, MK, and RK when using Dv2 pipeline. In addition, adaptive patch denoising generally results in the lowest median percent error whereas eigenvalue shrinkage biases the results. On the other hand, adaptive patch denoising with eigenvalue shrinkage has the fewest outliers and the least noise in maps (Figs. 6 and 7). Thus, while adaptive patch denoising gives the most accurate median value after omitting outliers, we set adaptive patch denoising with eigenvalue shrinkage as the default denoising in Dv2 as it removes the most noise and results in the least voxels due to outliers.

Alternatively, smoothing can be applied to reduce noise, as demonstrated in Figures 2 and 8. Smoothing appears to reduce noise in clinical dMRI maps (Fig. 2) but does not perform as well as applying denoising in Dv2. Similarly in noise phantoms (Fig. 8), smoothing reduces noise at low SNR for certain parameters, but adaptive patch denoising and Rician bias correction perform better, and smoothing may actually be more harmful for SNR>20. Thus, smoothing is a reasonable option if denoising cannot be applied but will not yield optimal results, and ideally should be preceded by denoising.

Dv2’s modified Gibbs removal method, RPG, was also evaluated using ground truth phantoms. RPG targets ringing due to partial Fourier, particularly for 7/8 and 6/8 partial Fourier. Lee et al. have demonstrated, correcting for 5/8 partial Fourier may result in unreasonably smoothed maps (Lee et al., 2021). Mean percent error in manually drawn ROIs (targeting the ringing artifact) on Shepp-Logan phantom with 6/8 partial Fourier shows improved accuracy using RPG compared to SuShi (Fig. 9). This can be visually verified in percent error maps (Fig. 9A). Hence, for clinical dMRI with similar partial Fourier acquisition, we verify that it would be valuable to use RPG to effectively remove Gibbs ringing and improve accuracy in parameter maps.

Further improvements to yield more accurate parameter maps may be to adjust the shrinkage when denoising to minimize bias, consider signal drift, and to deal with remaining black voxels. Signal drift has been found to cause a global decrease in signal intensity due to temporal scanner instability and can vary from scanner to scanner (Vos et al., 2017). Vos et al. investigated the effects of signal drift in dMRI data and proposed to fit a nonlinear model using interspersed *b* = 0 images from the scan to interpolate and calculate correction factors for the images. This is a similar correction to DESIGNER-v2’s B0 normalization which can be adjusted and carried over to correct for signal drift. Evaluating signal drift in our clinical dMRI and incorporating this correction in our pipeline may yield more accurate parameter estimates. Although the DESIGNER-v2 pipeline greatly reduces the number of outlier voxels, for the diffusion acquisition protocol employed in our study, we still cannot avoid problematic black voxels in kurtosis maps just from preprocessing. The remaining black voxels may be eliminated in the tensor fitting step via a combination of outlier detection and correction, smoothing, and robust fitting (Henriques, Jespersen, et al., 2021).

This work focused on evaluating denoising and Gibbs removal as additional components in image preprocessing pipelines. We conclude that the addition of denoising and Gibbs ringing correction improves the performance of the preprocessing pipeline. Similar findings were recently reported by (Cieslak et al., 2024) albeit their study was limited to denoising only. In its essence, we demonstrate the impact of improving individual preprocessing steps to maximize the performance of the whole pipeline, without ranking or quantifying the relative contribution of a single preprocessing step on the overall performance. Hence, our results do not suggest that the use of denoising or Gibbs ringing removal lowers the importance of other image preprocessing steps such as motion correction. Indeed, for example, dMRI datasets in this study were initially preprocessed on servers with different versions of FSL (v5.0.8 versus v6.0.5), giving us inconsistent results and prompting us to reprocess all data with FSL v6.0.5 on the same server. Figure S12 shows the importance of using an updated eddy and motion correction version by updating FSL as resulting maps were suboptimal regardless of denoising and Gibbs removal.

Various recent pipelines (Cai et al., 2021; Cieslak et al., 2021; Cui et al., 2013; Irfanoglu et al., 2017), including DESIGNER (Dv1 and Dv2), are similar in that they target the correction of artifact-specific approaches and are agnostic to further post-processing. Thus, highlighted advances, including RPG, might represent relevant development for any other pipeline. Moreover, DESIGNER-v2 is developed and validated on a large cohort of clinical data that represent the realistic challenges and variability of clinical radiology. Therefore, we believe that DESIGNER-v2 is well-equipped to bridge the gap between clinical and research applications of neuroimaging.

DESIGNER is available at https://github.com/NYU-DiffusionMRI/DESIGNER-v2 where instructions are available for using DESIGNER with Docker or with installation via pip. Dependencies for running the pipelines in this paper include MRtrix3 (https://www.mrtrix.org), FMRIB Software Library (FSL (v6.0.5 was used for this study), https://fsl.fmrib.ox.ac.uk/fsl/fslwiki/FSL), and Python. We recommend making sure FSL versions are updated when preprocessing data for studies as older versions of FSL may not perform as well and mixing FSL versions can give inconsistent results. One way to ensure consistent pipeline is used would be to run DESIGNER using Docker. DESIGNER comes with detailed documentation (https://nyu-diffusionmri.github.io/DESIGNER-v2) for each available preprocessing option, so users only need a basic understanding of dMRI data.

## 5 Conclusion

The updated DESIGNER (Dv2) pipeline provides accurate and robust diffusion parameters for clinical research studies. It is designed to be used on dMRI scans acquired by standard multi-shell dMRI clinical protocols with common artifacts. DESIGNER-v2 is flexible enough to allow steps to be left out or adjusted to cater to a wider range of images. The standard pipeline consists of: (a) MPPCA adaptive patch denoising with eigenvalue shrinkage, (b) RPG (PF < 1) or SuShi (Kellner et al., 2016) (no PF) correction for Gibbs, (c) EPI distortion correction, (d) Eddy current and motion correction, (e) B0 normalization, and (f) Rician bias correction. Our study shows the benefit of using the DESIGNER-v2 pipeline on diffusion MRI with common artifacts as no preprocessing or minimal preprocessing may result in many outliers and unreliable diffusion parameters values.

## Supplementary Material

Supplementary Material

## Data Availability

dMRI data are available upon request. DESIGNER is available at https://github.com/NYU-DiffusionMRI/DESIGNER-v2 as a preprocessing tool for diffusion MRI.
