# Supplementary material for: Optimization and validation of the DESIGNER preprocessing pipeline for clinical diffusion MRI in white matter aging
Source: Imaging Neurosci (Camb). 2024 Apr 8;2:imag-2-00125. doi: 10.1162/imag_a_00125 (PMC12247605; doi:10.1162/imag_a_00125)
Supplement: Supplementary Material [file imag_a_00125-supp.pdf]

SUPPLEMENTARY

Table S1. Adjusted R^2 from quadratic and linear fit of age correlation with DTI and DKI parameters in white matter regions (median value) using no preprocessing pipeline, E+M, DESIGNER-v1, and DESIGNER-v2 preprocessing pipeline (Prisma, TE = 70ms, N = 142). Adjusted R^2s from quadratic fit are larger than adjusted R^2s from linear fit for all age associations. Bolded adjusted R^2 indicates statistically significant age association with adjusted R^2 > 0.1. Cell color-scale from light to dark blue indicates lowest to highest adjusted R^2.

			MD	RD	AD	FA	MK	RK	AK
PLIC	None	Quadratic	0.00	0.07	**0.15**	**0.12**	0.01	0.01	0.02
		Linear	0.00	0.06	**0.14**	**0.11**	-0.01	-0.01	0.02
	E+M	Quadratic	0.06	0.05	**0.16**	**0.10**	0.02	0.04	0.08
		Linear	0.03	0.05	**0.15**	**0.10**	0.02	0.04	0.08
	DESIGNER-v1	Quadratic	0.03	0.04	**0.12**	0.08	0.03	0.08	0.08
		Linear	0.02	0.03	**0.11**	0.08	0.03	0.08	0.07
	DESIGNER-v2	Quadratic	0.03	0.05	0.10	0.09	0.02	0.08	0.10
		Linear	0.01	0.04	0.09	0.09	0.02	0.08	0.09
SCC	None	Quadratic	0.01	0.05	0.02	0.06	0.01	0.02	0.08
		Linear	0.01	0.05	0.01	0.06	0.01	0.02	0.05
	E+M	Quadratic	0.00	0.03	0.01	0.05	0.00	0.00	**0.13**
		Linear	0.00	0.03	0.01	0.05	0.00	0.00	**0.11**
	DESIGNERv1	Quadratic	0.01	0.04	0.00	0.05	0.00	-0.01	**0.16**
		Linear	0.01	0.04	0.00	0.05	-0.01	-0.01	**0.11**
	DESIGNER-v2	Quadratic	0.04	0.09	0.00	0.08	0.00	0.00	**0.16**
		Linear	0.04	0.08	0.00	0.08	-0.01	0.00	**0.11**
GCC	None	Quadratic	**0.17**	**0.21**	0.07	**0.20**	0.01	0.01	0.02
		Linear	**0.13**	**0.19**	0.01	**0.19**	-0.01	-0.01	0.02
	E+M	Quadratic	**0.19**	**0.22**	0.05	**0.18**	0.03	0.01	0.00
		Linear	**0.14**	**0.20**	-0.01	**0.18**	0.01	0.00	0.00
	DESIGNER-v1	Quadratic	**0.19**	**0.24**	0.02	**0.22**	0.07	0.06	0.01
		Linear	**0.16**	**0.22**	-0.01	**0.21**	0.04	0.03	-0.01
	DESIGNER-v2	Quadratic	**0.22**	**0.29**	0.01	**0.24**	**0.11**	0.08	0.00
		Linear	**0.18**	**0.26**	0.00	**0.23**	0.07	0.06	-0.01
ACR	None	Quadratic	**0.24**	**0.34**	0.00	**0.27**	0.05	0.03	0.01
		Linear	**0.21**	**0.31**	0.00	**0.26**	0.03	0.02	0.00
	E+M	Quadratic	**0.23**	**0.32**	0.00	**0.29**	**0.12**	**0.13**	0.00
		Linear	**0.20**	**0.30**	-0.01	**0.28**	**0.11**	**0.12**	-0.01
	DESIGNER-v1	Quadratic	**0.23**	**0.32**	-0.01	**0.29**	**0.14**	**0.19**	0.01
		Linear	**0.21**	**0.29**	-0.01	**0.26**	**0.13**	**0.17**	0.00
	DESIGNER-v2	Quadratic	**0.26**	**0.33**	0.00	**0.29**	**0.16**	**0.23**	0.01
		Linear	**0.23**	**0.30**	-0.01	**0.25**	**0.14**	**0.21**	0.00

Table S2. Bonferroni adjusted P-values for age correlation with DTI and DKI parameters in white matter regions (median value) using no preprocessing pipeline, E+M, DESIGNER-v1, and DESIGNER-v2 preprocessing pipeline (Prisma, TE = 70ms, N = 142).

		MD	RD	AD	FA	MK	RK	AK
PLIC	None	1.000	0.037	<0.001	<0.001	1.000	1.000	1.000
	E+M	0.051	0.104	<0.001	0.002	1.000	0.318	0.014
	DESIGNER-v1	0.615	0.416	<0.001	0.007	0.489	0.012	0.016
	DESIGNER-v2	0.681	0.174	0.003	0.005	1.000	0.010	0.003
SCC	None	1.000	0.128	1.000	0.061	1.000	1.000	0.014
	E+M	1.000	0.487	1.000	0.130	1.000	1.000	<0.001
	DESIGNER-v1	1.000	0.297	1.000	0.119	1.000	1.000	<0.001
	DESIGNER-v2	0.223	0.006	1.000	0.008	1.000	1.000	<0.001
GCC	None	<0.001	<0.001	0.019	<0.001	1.000	1.000	1.000
	E+M	<0.001	<0.001	0.173	<0.001	0.543	1.000	1.000
	DESIGNER-v1	<0.001	<0.001	1.000	<0.001	0.025	0.066	1.000
	DESIGNER-v2	<0.001	<0.001	1.000	<0.001	0.001	0.008	1.000
ACR	None	<0.001	<0.001	1.000	<0.001	0.174	0.513	1.000
	E+M	<0.001	<0.001	1.000	<0.001	<0.001	<0.001	1.000
	DESIGNER-v1	<0.001	<0.001	1.000	<0.001	<0.001	<0.001	1.000
	DESIGNER-v2	<0.001	<0.001	1.000	<0.001	<0.001	<0.001	1.000

Table S3. Bonferroni adjusted P-values for age correlation with DTI and DKI parameters in white matter regions (median value) using no preprocessing pipeline, E+M, DESIGNER-v1, and DESIGNER-v2 preprocessing pipeline (Prisma, TE = 95ms, N = 120).

		MD	RD	AD	FA	MK	RK	AK
PLIC	None	1.000	1.000	0.388	1.000	0.855	1.000	0.282
	E+M	1.000	1.000	1.000	1.000	0.948	1.000	0.169
	DESIGNER-v1	1.000	1.000	1.000	1.000	0.064	0.134	0.110
	DESIGNER-v2	1.000	1.000	1.000	1.000	0.009	0.055	0.076
SCC	None	1.000	1.000	0.662	1.000	1.000	1.000	0.252
	E+M	1.000	1.000	1.000	1.000	1.000	1.000	0.176
	DESIGNER-v1	1.000	1.000	1.000	1.000	1.000	1.000	0.023
	DESIGNER-v2	1.000	0.107	1.000	0.037	0.895	1.000	0.036
GCC	None	0.004	<0.001	0.943	<0.001	1.000	1.000	1.000
	E+M	0.052	0.001	0.012	<0.001	0.209	1.000	1.000
	DESIGNER-v1	0.059	<0.001	0.139	<0.001	<0.001	0.001	1.000
	DESIGNER-v2	0.001	<0.001	0.126	<0.001	<0.001	<0.001	1.000
ACR	None	<0.001	<0.001	1.000	<0.001	0.020	0.147	1.000
	E+M	<0.001	<0.001	1.000	<0.001	0.001	0.009	1.000
	DESIGNER-v1	<0.001	<0.001	1.000	<0.001	<0.001	<0.001	0.513
	DESIGNER-v2	<0.001	<0.001	1.000	<0.001	<0.001	<0.001	0.441

Table S4. Adjusted R^2 for age correlation with DTI and DKI parameters in white matter regions (median value) using no preprocessing pipeline, E+M, DESIGNER-v1, and DESIGNER-v2 preprocessing pipeline (Prisma, TE = 95ms, N = 120). Bolded adjusted R^2 indicates statistical significance with adjusted R^2 > 0.1. Cell color-scale from light to dark blue indicates lowest to highest adjusted R^2.

		MD	RD	AD	FA	MK	RK	AK
PLIC	None	0.01	-0.01	0.04	0.00	0.03	0.03	0.05
	E+M	0.00	-0.01	0.01	0.00	0.03	0.02	0.05
	DESIGNER-v1	0.01	-0.01	0.01	0.00	0.07	0.06	0.06
	DESIGNER-v2	0.00	0.00	0.01	0.02	0.10	0.07	0.07
SCC	None	0.01	0.02	0.03	0.02	0.02	0.01	0.05
	E+M	-0.01	0.01	0.01	0.02	0.02	0.01	0.05
	DESIGNER-v1	-0.01	0.01	0.02	0.03	0.02	0.02	0.08
	DESIGNER-v2	0.00	0.06	0.02	0.08	0.03	0.03	0.08
GCC	None	**0.11**	**0.21**	0.03	**0.25**	0.00	0.00	0.02
	E+M	0.07	**0.13**	0.09	**0.20**	0.05	0.01	0.00
	DESIGNER-v1	0.07	**0.19**	0.06	**0.26**	**0.14**	**0.13**	0.02
	DESIGNER-v2	**0.12**	**0.25**	0.06	**0.31**	**0.17**	**0.18**	0.03
ACR	None	**0.24**	**0.29**	0.02	**0.26**	0.09	0.06	0.00
	E+M	**0.22**	**0.30**	0.01	**0.28**	**0.13**	0.10	0.03
	DESIGNER-v1	**0.22**	**0.31**	0.00	**0.30**	**0.23**	**0.29**	0.04
	DESIGNER-v2	**0.21**	**0.32**	0.00	**0.30**	**0.23**	**0.29**	0.04

Table S5. Bonferroni adjusted P-values for age correlation with DTI and DKI parameters in white matter regions (median value) using no preprocessing pipeline, E+M, DESIGNER-v1, and DESIGNER-v2 preprocessing pipeline (Skyra, TE = 95ms, N = 262).

		MD	RD	AD	FA	MK	RK	AK
PLIC	None	1.000	0.201	1.000	0.526	1.000	1.000	<0.001
	E+M	0.920	0.006	1.000	0.001	1.000	1.000	<0.001
	DESIGNER-v1	1.000	0.041	1.000	0.007	1.000	1.000	<0.001
	DESIGNER-v2	1.000	0.026	1.000	0.002	0.427	1.000	<0.001
SCC	None	1.000	1.000	1.000	1.000	1.000	1.000	0.005
	E+M	1.000	0.805	1.000	0.707	0.015	0.089	<0.001
	DESIGNER-v1	1.000	1.316	1.000	1.000	0.114	0.084	<0.001
	DESIGNER-v2	0.500	0.067	1.000	0.067	0.371	0.350	<0.001
GCC	None	<0.001	<0.001	0.227	<0.001	1.000	1.000	1.000
	E+M	<0.001	<0.001	0.361	<0.001	1.000	1.000	0.187
	DESIGNER-v1	<0.001	<0.001	0.549	<0.001	0.001	<0.001	0.104
	DESIGNER-v2	<0.001	<0.001	0.451	<0.001	<0.001	<0.001	0.196
ACR	None	<0.001	<0.001	1.000	<0.001	0.370	0.931	0.822
	E+M	<0.001	<0.001	1.000	<0.001	0.016	0.004	0.226
	DESIGNER-v1	<0.001	<0.001	1.000	<0.001	<0.001	<0.001	0.613
	DESIGNER-v2	<0.001	<0.001	1.000	<0.001	<0.001	<0.001	0.213

Table S6. Adjusted R^2 for age correlation with DTI and DKI parameters in white matter regions (median value) using no preprocessing pipeline, E+M, DESIGNER-v1, and DESIGNER-v2 preprocessing pipeline (Skyra, TE = 95ms, N = 262). Bolded adjusted R^2 indicates statistical significance with adjusted R^2 > 0.1. Cell color-scale from light to dark blue indicates lowest to highest adjusted R^2.

		MD	RD	AD	FA	MK	RK	AK
PLIC	None	0.01	0.02	0.01	0.02	0.00	0.00	0.09
	E+M	0.01	0.05	0.00	0.06	0.00	0.00	**0.12**
	DESIGNER-v1	0.01	0.03	0.00	0.05	0.01	0.00	0.07
	DESIGNER-v2	0.01	0.04	0.00	0.05	0.02	0.00	0.08
SCC	None	0.00	0.00	0.01	0.01	0.00	0.00	0.05
	E+M	0.00	0.01	0.01	0.02	0.04	0.03	0.09
	DESIGNER-v1	0.01	0.01	0.00	0.01	0.03	0.03	**0.12**
	DESIGNER-v2	0.02	0.03	0.00	0.03	0.02	0.02	0.10
GCC	None	**0.11**	**0.18**	0.02	**0.19**	0.00	0.00	0.01
	E+M	**0.10**	**0.20**	0.02	**0.20**	0.00	0.00	0.02
	DESIGNER-v1	**0.10**	**0.20**	0.02	**0.22**	0.06	0.07	0.03
	DESIGNER-v2	**0.15**	**0.24**	0.02	**0.25**	0.08	0.08	0.02
ACR	None	**0.17**	**0.26**	0.01	**0.22**	0.02	0.01	0.01
	E+M	**0.17**	**0.25**	0.00	**0.24**	0.04	0.05	0.02
	DESIGNER-v1	**0.16**	**0.25**	0.01	**0.23**	0.09	**0.16**	0.02
	DESIGNER-v2	**0.17**	**0.26**	0.00	**0.24**	**0.10**	**0.17**	0.02

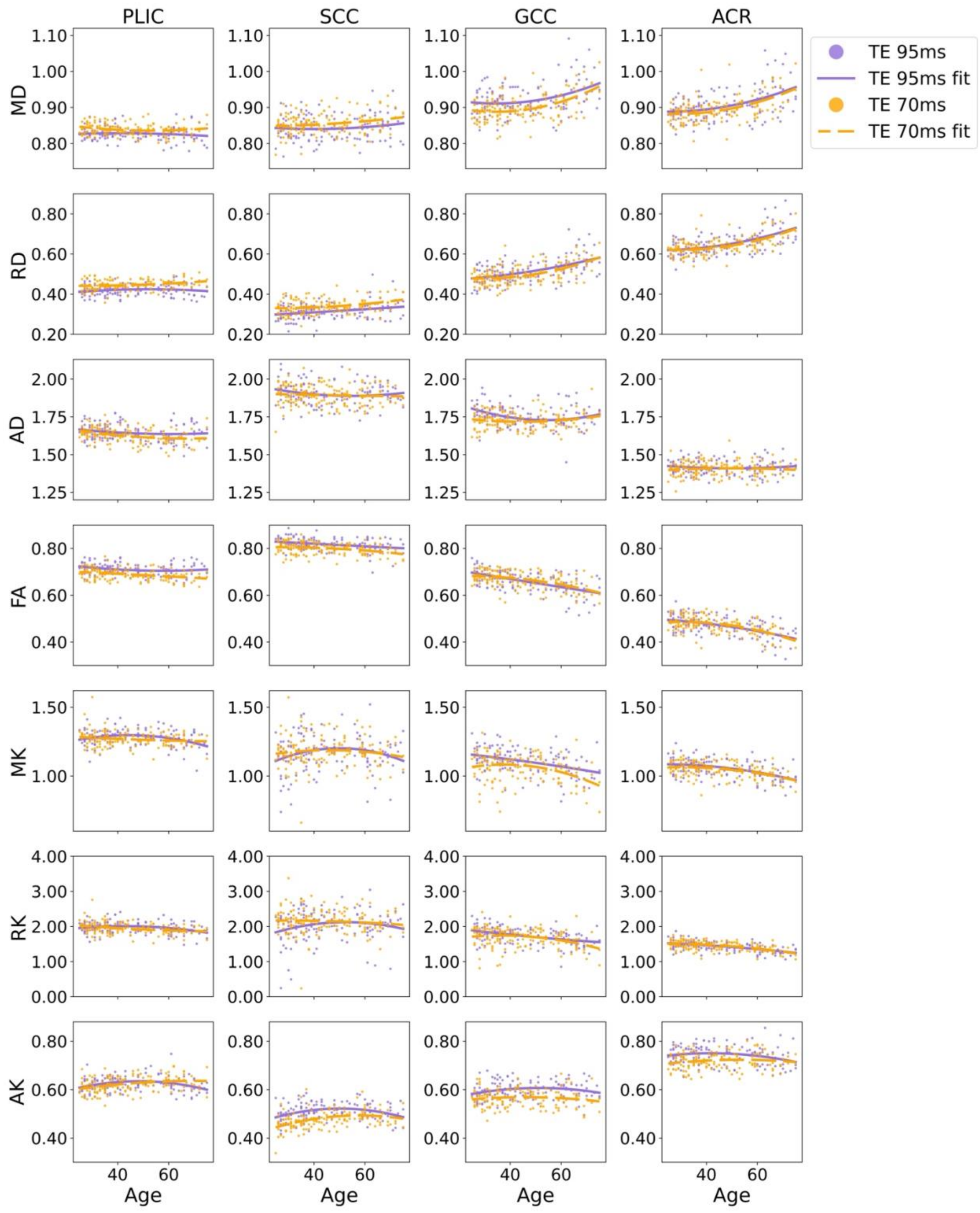

Figure S1. Age correlation with quadratic fits for DTI and DKI parameters in white matter ROIs (median) from DESIGNER-v2 pipeline for normal subjects with TE=95ms (N=120, Prisma) and TE=70ms (N=142, Prisma).

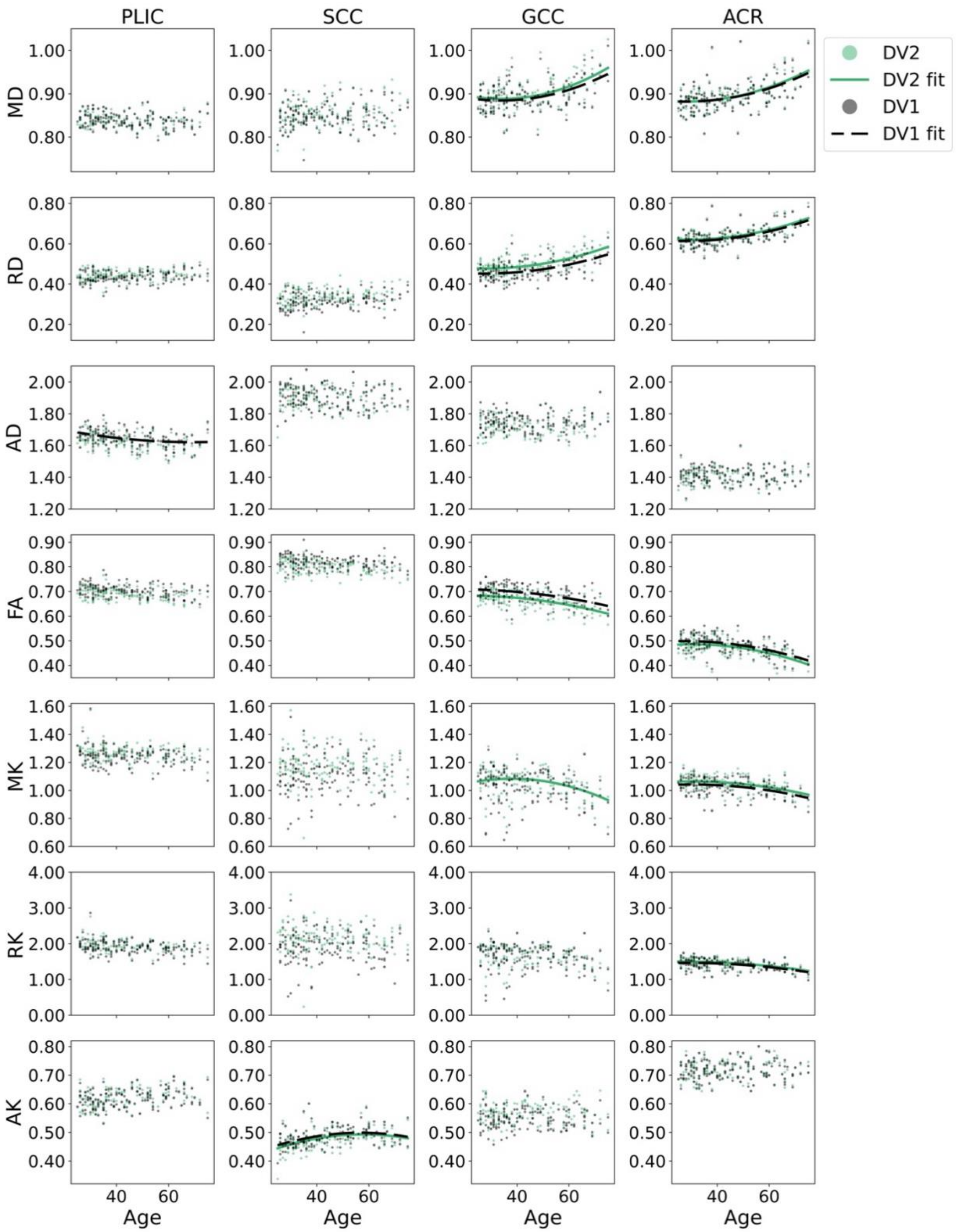

Figure S2. Age correlation with DTI and DKI parameters in white matter ROIs (median) from DESIGNER-v2 and DESIGNER-v1 pipeline of 142 healthy subjects (Prisma, TE = 70ms). Quadratic fits were plotted for statistically significant correlations with adjusted R^2 > 0.1 only.

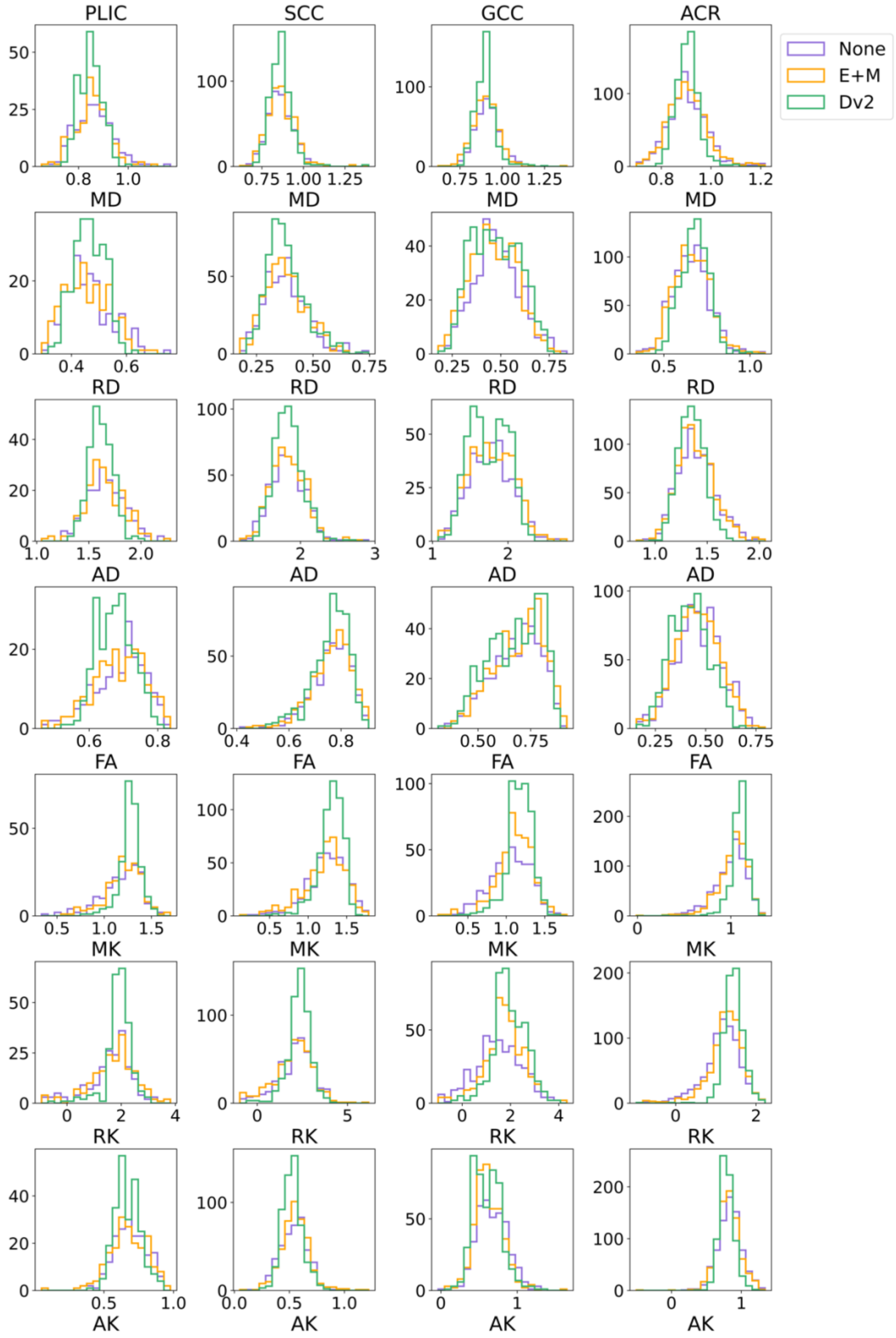

Figure S3. Probability density of DTI and DKI parameters after omitting outliers in white matter ROIs from data preprocessed with DESIGNER-v2, E+M, or no preprocessing pipeline for a healthy 60-year-old female.

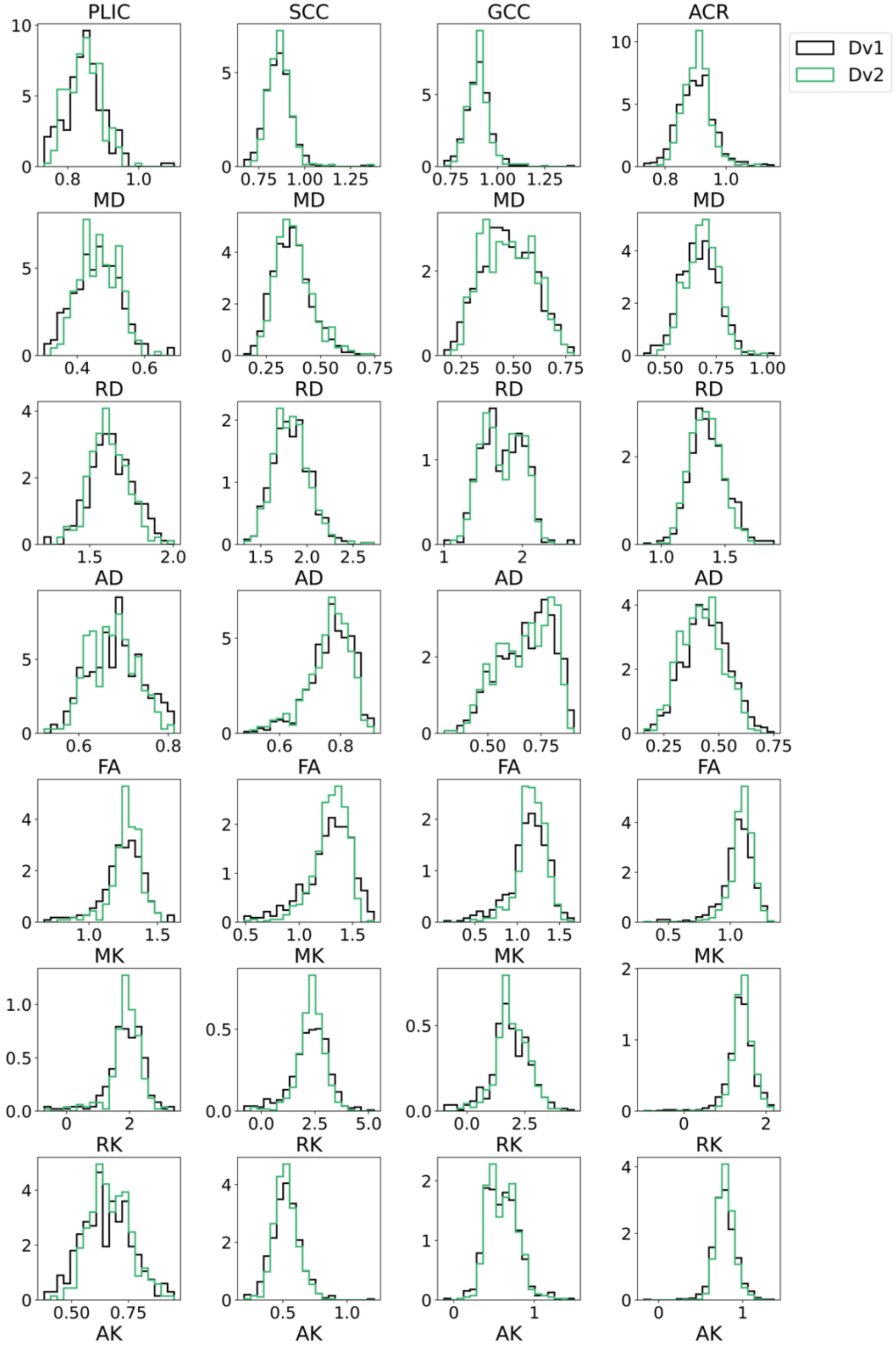

Figure S4. Probability density of DTI and DKI parameters after omitting outliers in white matter ROIs from data preprocessed with DESIGNER-v2 or DESIGNER-v1 pipeline, for a healthy 60-year-old female.

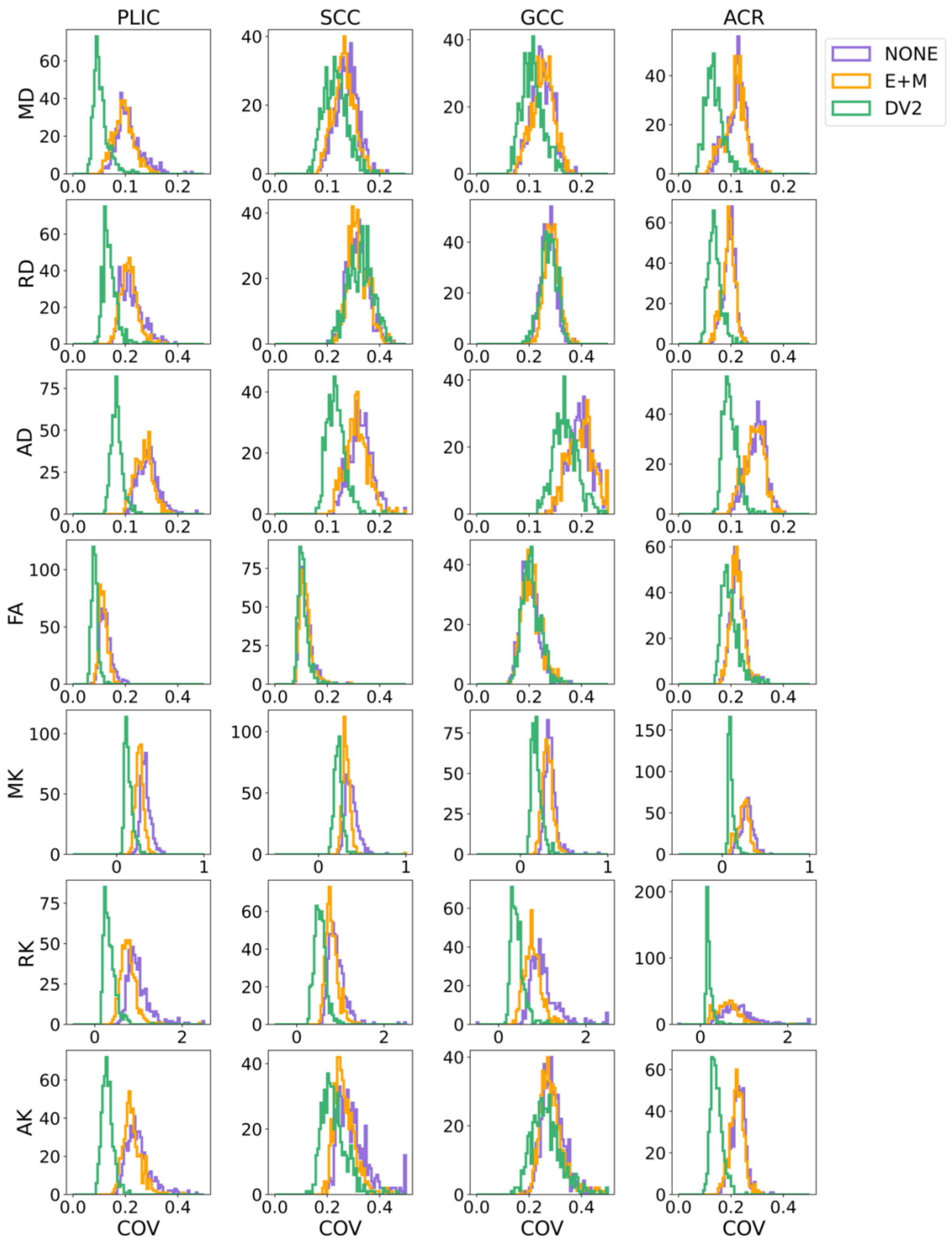

Figure S5. Histogram of coefficient of variation (COV=standard deviation/mean of ROI) after omitting outliers in white matter ROIs of DTI and DKI maps from DESIGNER-v2, E+M, or no preprocessing pipeline for 524 healthy subjects.

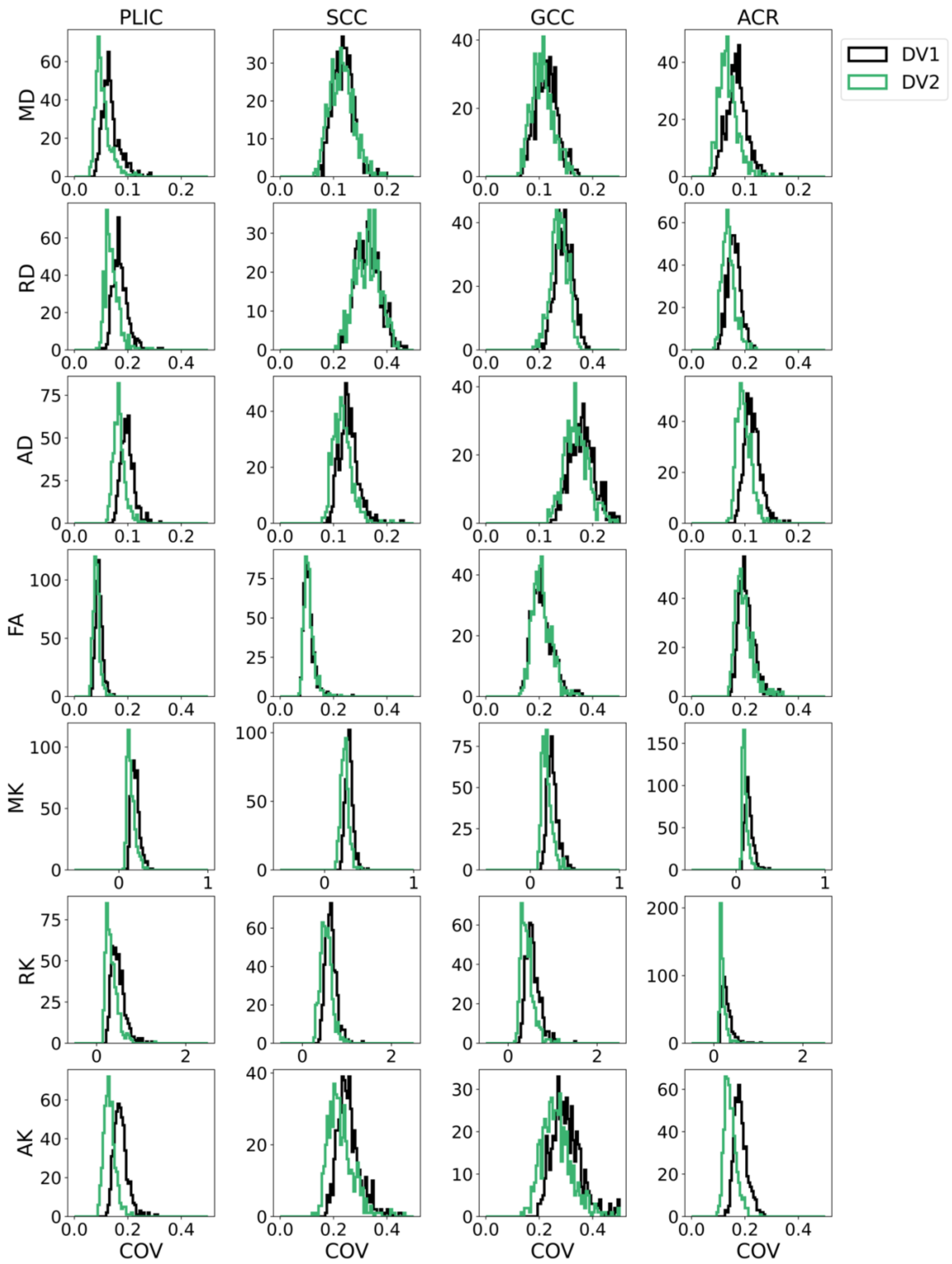

Figure S6. Histogram of coefficient of variation (COV=standard deviation/mean of ROI) after omitting outliers in white matter ROIs of DTI and DKI maps from DESIGNER-v2 and DESIGNER-v1 pipeline for 524 healthy subjects.

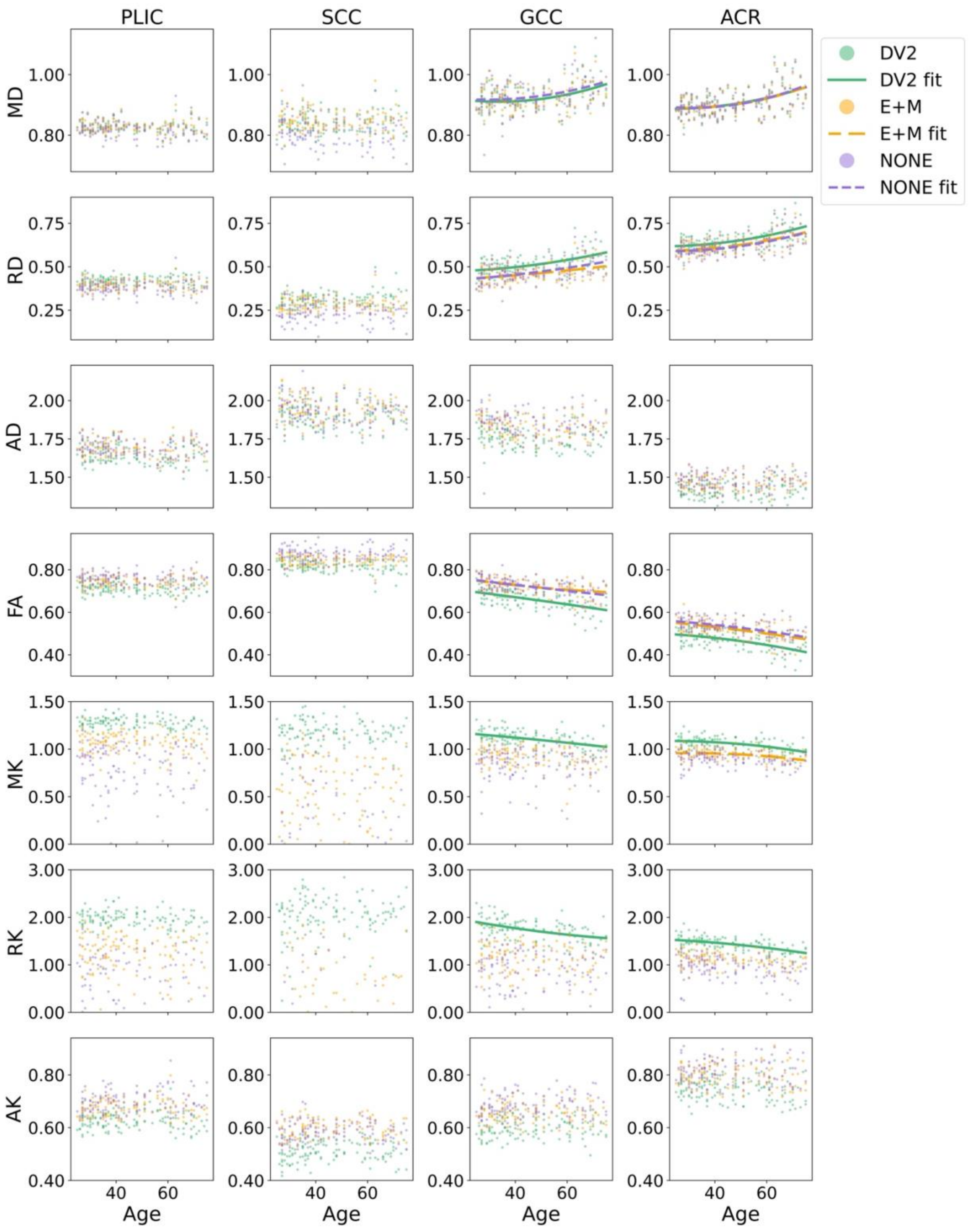

Figure S7. Age correlation with DTI and DKI parameters in white matter ROIs (median) from DESIGNER-v2 pipeline, E+M, or not preprocessing pipeline of 120 healthy subjects (Prisma, TE = 95ms). Quadratic fits were plotted for statistically significant correlations with adjusted R^2 > 0.1 only. MK and RK plots are zoomed in so not all datapoints are visible.

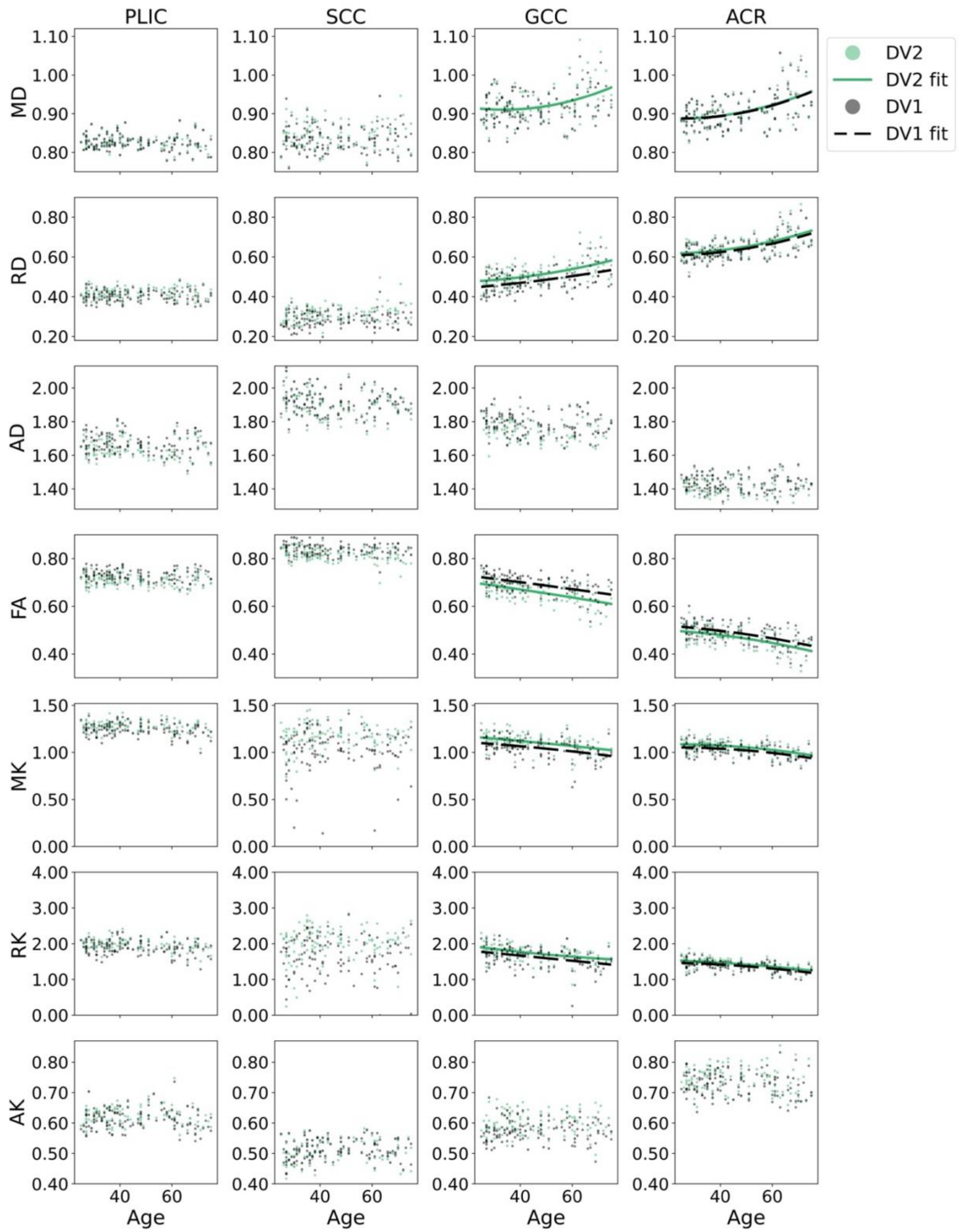

Figure S8. Age correlation with DTI and DKI parameters in white matter ROIs (median) from DESIGNER-v2 and DESIGNER-v1 pipeline of 120 healthy subjects (Prisma, TE = 95ms). Quadratic fits were plotted for statistically significant correlations with adjusted R^2 > 0.1 only.

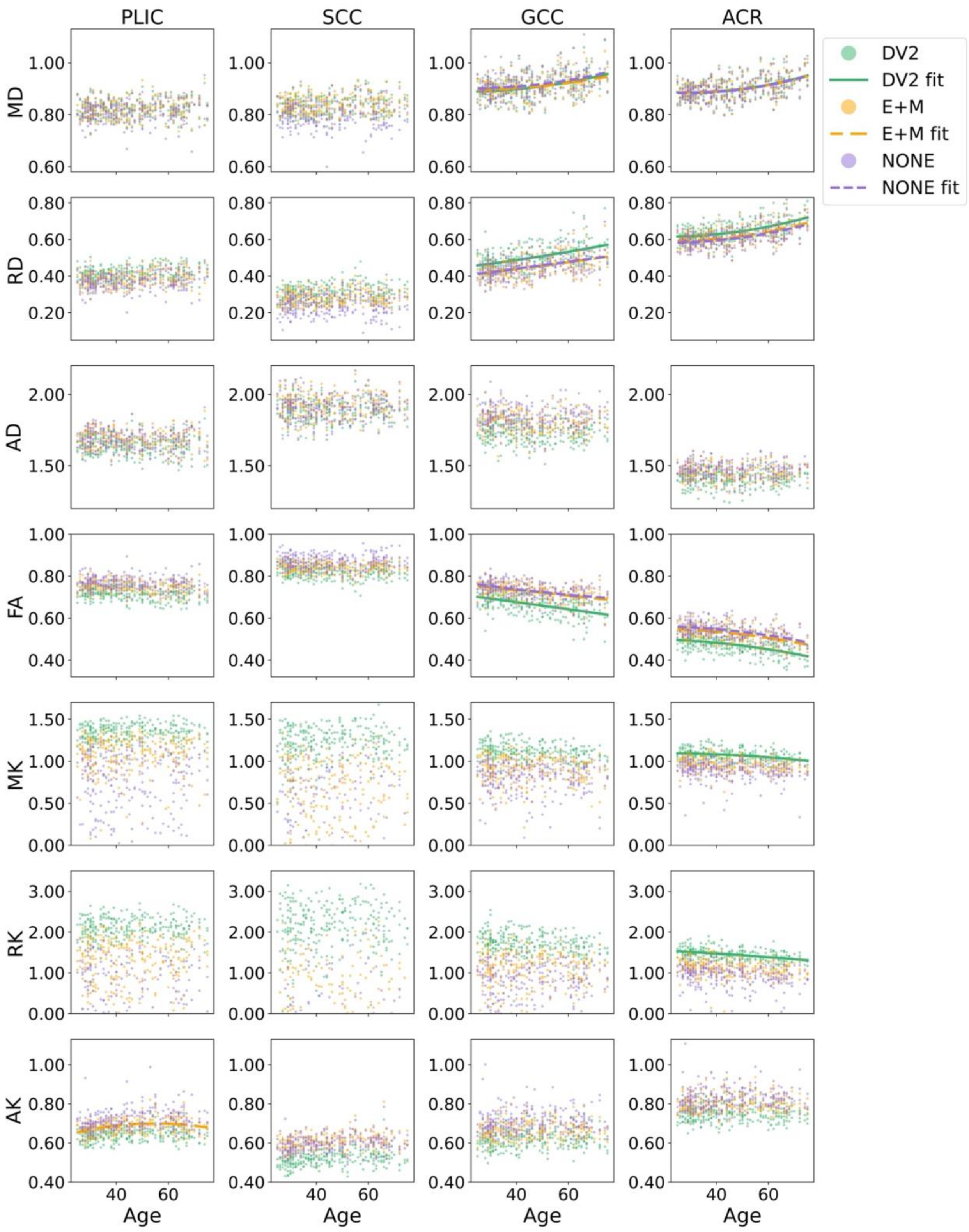

Figure S9. Age correlation with DTI and DKI parameters in white matter ROIs (median) from DESIGNER-v2 pipeline, E+M, or not preprocessing pipeline of 262 healthy subjects (Skyra, TE = 95ms). Quadratic fits were plotted for statistically significant correlations with adjusted $R^2 > 0.1$ only. MK and RK plots are zoomed in so not all datapoints are visible.

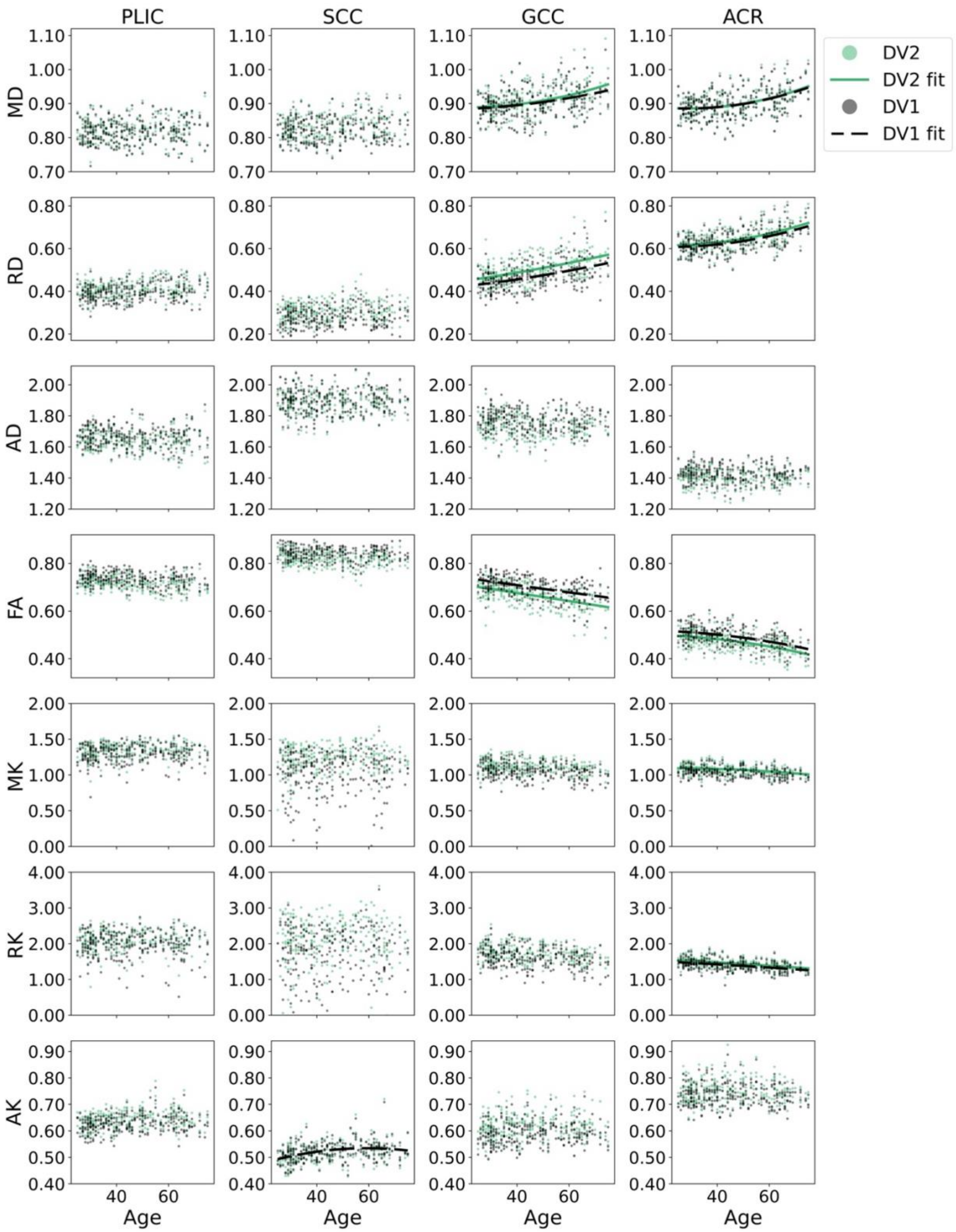

Figure S10. Age correlation with DTI and DKI parameters in white matter ROIs (median) from DESIGNER-v2 and DESIGNER-v1 pipeline of 262 healthy subjects (Skyra, TE = 95ms). Quadratic fits were plotted for statistically significant correlations with adjusted R^2 > 0.1 only.

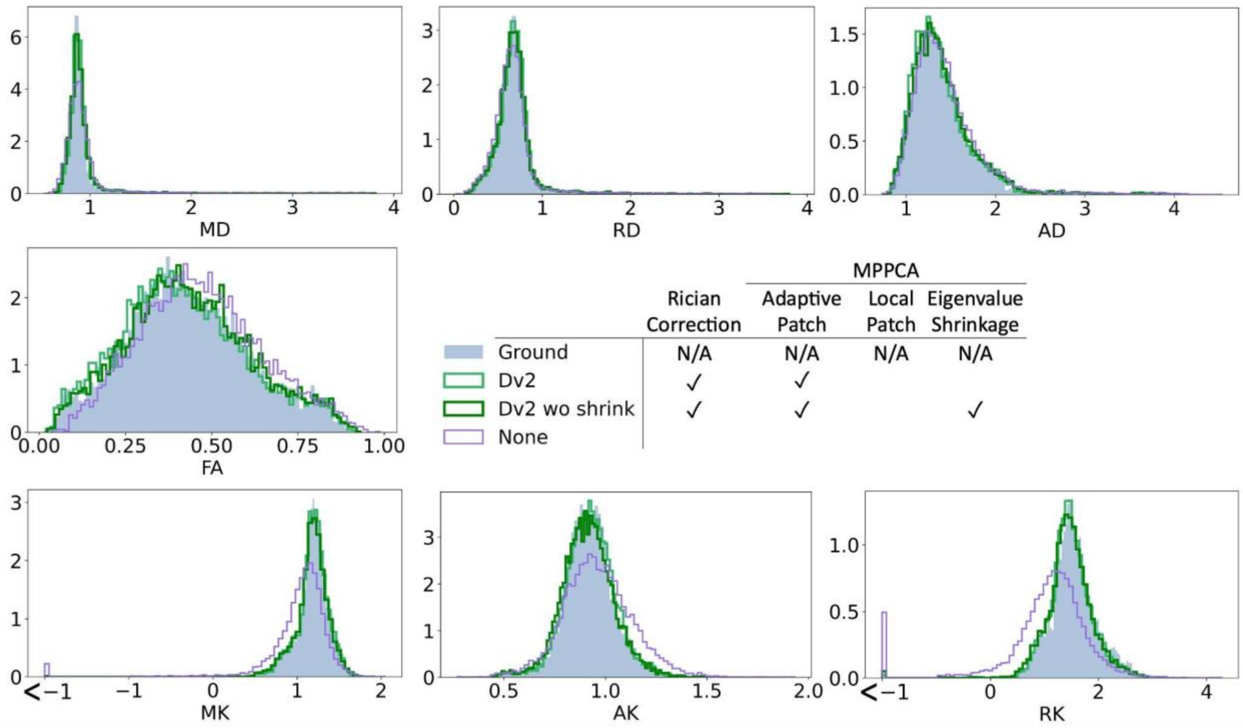

Figure S11. Probability density of DTI and DKI parameters in white matter of ground truth HCP phantom and HCP noise phantom (SNR 20) after adaptive patch denoising with eigenvalue shrinkage (with Rician bias correction) and adaptive patch denoising without eigenvalue shrinkage (with Rician bias correction).

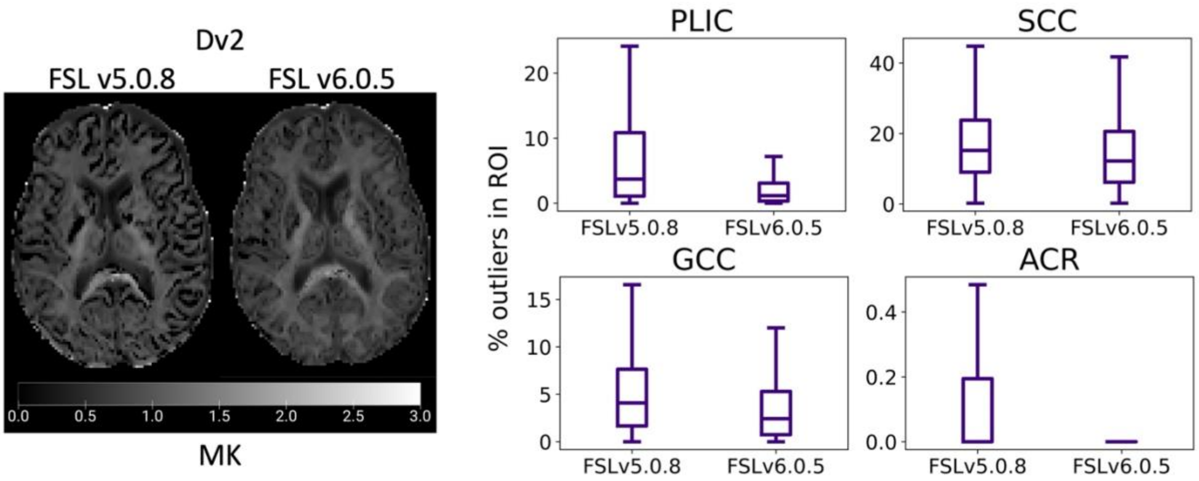

Figure S12. Left: MK maps from a healthy 69-year-old female after preprocessing with DESIGNER-v2 using different FSL versions (v5.0.8 and v6.0.5) for motion and eddy current correction. Right: Box plots of percent outliers (100*number of outliers in ROI/number of voxels in ROI) in each WM ROI of the dMRI parameter maps of 524 subjects based on DESIGNER-v2 pipeline using different FSL versions. One-way ANOVA showed percent outliers in each of the four ROIs were significantly different between FSL versions except in ACR.
